# Early prediction of gestational diabetes mellitus using deep learning applied to routinely collected clinical data

**DOI:** 10.3389/fendo.2026.1941799

**Published:** 2026-10-05

**Authors:** Gabriel Cubillos, Max Monckeberg, Sebastian E. Illanes, Claudio A. Perez

**Affiliations:** 1 Department of Electrical Engineering, Universidad de Chile, Santiago, Chile; 2 IMPACT, Center of Interventional Medicine for Precision and Advanced Cellular Therapy, Santiago, Chile; 3 Department of Obstetrics and Gynecology and Laboratory of Reproductive Biology, Faculty of Medicine, Universidad de los Andes, Santiago, Chile

**Keywords:** clinical decision support, convolutional neural networks, deep learning models, early risk prediction, explainable artificial intelligence, genetic algorithms, gestational diabetes mellitus, risk stratification

## Abstract

**Background:**

Prediction of gestational diabetes mellitus (GDM) in early pregnancy enables timely, targeted interventions to reduce risks for both the mother and fetus. We aimed to improve early GDM prediction and clinical risk stratification using a novel deep learning (DL) approach that transforms tabular clinical data into images using a genetic algorithm.

**Methods:**

A dataset of 1,611 pregnancies was used. The dataset contained routinely collected early-pregnancy data, including clinical measurements, blood tests, and medical history. A novel method to transform tabular data into images was applied to enable the use of deep learning models for GDM prediction. A genetic algorithm optimized the spatial arrangement of variables during image generation by determining their spatial positions. Interpretability was assessed using gradient-weighted class activation mapping (Grad-CAM) to identify variables the model preferentially attended to for positive and negative predictions.

**Results:**

Multiple model configurations were evaluated by varying the spatial arrangement of variables within the transformation. Prioritizing high sensitivity, one model achieved an accuracy of 0.86, sensitivity of 0.82, specificity of 0.87, and an AUC-ROC of 0.83. Another model achieved an accuracy of 0.79, sensitivity of 0.92, specificity of 0.77, and an AUC-ROC of 0.85. Compared with previously published machine learning models using the same dataset, our approach demonstrated improved performance. Our best models increased specificity by an average of 13.93 percentage points compared with traditional ML models at matched sensitivity levels, thereby reducing false-positive classifications that may lead to additional clinical evaluation. Grad-CAM analysis revealed that attention patterns of the model aligned with established clinical risk factors for GDM, with first trimester fasting glycemia and parity preferentially attended for positive predictions and maternal weight for negative predictions.

**Conclusions:**

This study demonstrates that transforming tabular clinical data into image representations, optimized using a genetic algorithm, improves early GDM prediction. Representing tabular variables as images enables more effective application of DL models, which have demonstrated enhanced pattern recognition in image-based tasks compared with traditional machine learning methods. Performance improvements over state-of-the-art approaches were achieved. Accurate DL models can be developed using a limited number of input variables, supporting early identification of women at increased risk of GDM and facilitating timely clinical management.

## Introduction

Gestational diabetes mellitus (GDM) is a condition defined as glucose intolerance of varying severity that begins or is first recognized during pregnancy and does not meet the diagnostic criteria for overt diabetes mellitus (1). Globally, an estimated 15.6% of pregnancies are affected by GDM (1), making it one of the most prevalent metabolic complications of pregnancy and a growing public health concern amid rising rates of obesity and sedentary lifestyles worldwide. GDM is associated with an increased risk of several diseases in both the mother and the fetus and contributes substantially to maternal and neonatal morbidity (2–4). Possible effects in the mother include developing Type 2 Diabetes Mellitus, depression, and hepatic, renal, and cardiovascular diseases (5–7). The fetus faces elevated risk of developing adverse outcomes such as insulin resistance, macrosomia, preterm birth, respiratory distress, the need for intensive care unit admission, or stillbirth (5–7). These multigenerational consequences underscore the importance of GDM not only as an obstetric complication, but also as a major endocrine disorder and as a chronic disease risk factor with lifelong implications.

The diagnostic criteria for GDM lack global consensus and vary across different countries and regions (1). There is a growing trend toward the adoption of International Association of the Diabetes and Pregnancy Study Groups (IADPSG) criteria (8). This diagnostic approach involves an oral glucose tolerance test (OGTT), measuring plasma glucose at fasting and at one and two hours postprandially, using 75 g of glucose (8). However, this test is typically administered between gestational weeks 24 and 28, when many of the modifiable risk factors for GDM have already progressed beyond the period during which intervention is most effective.

Early diagnosis of GDM is essential because it allows timely interventions that reduce the complications mentioned above and improve maternal and fetal outcomes. Moreover, early screening provides an opportunity to implement preventive strategies that can decrease the likelihood of developing GDM. These measures include adopting a balanced diet, engaging in regular physical activity, and maintaining appropriate weight gain during pregnancy (1). Therefore, early preventive interventions can be achieved through simple measures that do not require complex treatments or medication. Importantly, these are low-cost, low-risk interventions that are feasible across diverse healthcare settings. Shifting the point of risk identification to the first trimester or early second trimester would substantially expand the window for such strategies, potentially preventing a significant proportion of GDM cases and their downstream consequences at a population level, thereby supporting earlier clinical decision-making and individualized prenatal care.

Machine learning (ML) models have been used in various fields, including control systems (9), finance, logistics, data visualization, and education, among others (10). ML has also been used in healthcare to support disease prediction, risk stratification, and clinical decision-making, including prediction of large-for-gestational-age infants (11), drug discovery, patient risk identification, disease diagnosis (12), and cancer prediction (13).

### State of the art in gestational diabetes mellitus prediction

Numerous models have been developed to predict GDM, as shown in our literature review (14–40). An Extreme Gradient Boosting (XGB) model incorporating maternal clinical and demographic variables, such as a history of GDM, family history of diabetes, previous adverse obstetric outcomes, maternal age, height, weight, ethnicity, and parity, demonstrated superior performance compared with other predictor–model combinations (14).

A Logistic Regression (LR) model using maternal age, pre-pregnancy body mass index (BMI), ethnicity, family history of diabetes, history of GDM, and adverse obstetric outcomes as input variables, was externally validated using data from multiple countries (15). Other study employed an LR model that included the triglyceride-glucose (TyG) index to assess the prediction of GDM (17). Another study analyzed 73 variables, including sociodemographic characteristics, clinical variables, and first-trimester laboratory measurements. The authors also evaluated a reduced model based on seven variables, including maternal age, previous GDM, family history of diabetes in a first-degree relative, multiple pregnancy, fasting plasma glucose (FPG), glycated hemoglobin (HbA1c), and triglycerides (TG). A deep neural network (DNN) and a LR model were applied to the full and reduced variable sets, respectively. Although the reduction in the number of variables resulted in decreased predictive performance, it was considered more clinically feasible and cost-effective (18).

An additional study considered maternal weight, height, age, ethnic origin, and family history of diabetes mellitus. This approach distinguishes nulliparous from parous women, with the latter group also accounting for a previous history of GDM. These factors are combined using a LR model (20). A similar approach is reported in a Turkish study, in which classification is stratified between nulliparous and primiparous women using an XGB model. The model incorporates maternal age, BMI, gravidity, parity, previous birth weight, smoking status, FPG, and family history of diabetes mellitus (32).

The aspartate aminotransferase–to–alanine transaminase ratio (AST/ALT), maternal age, pre-pregnancy BMI, waist circumference, glucose, TG, high-density lipoprotein cholesterol (HDL-C), and parity have also been used with a LR model to predict GDM (21). Maternal age, pre-pregnancy BMI, FPG, and TG were used to calculate the probability of GDM using a LR model in a study of Chinese women (27). Similarly, age, BMI, FPG, and family history of diabetes were incorporated into an LR model to predict GDM in another Chinese study (28). In addition, an LR model including age, history of GDM, pre-pregnancy BMI, white blood cell count, hematocrit, ALT, fasting blood glycemia (FBG), activated partial thromboplastin time (APTT), fibrinogen, and pregnancy-associated plasma protein A was shown to predict GDM in a Chinese population (31).

Pre-pregnancy BMI and selected blood test parameters, FPG, APTT, thrombin time, TG, low-density lipoprotein cholesterol (LDL-C), small dense low-density lipoprotein cholesterol (sdLDL-C), and apolipoprotein B, were used to predict GDM using an LR model (16). OGTT (1-hour and 2-hour postprandial), 2-hour postprandial insulin, and FBG measured in the first trimester, together with pregnancy BMI and maternal age, were used with a LR model (19). In another study, age, blood urea nitrogen, fibrinogen-to-albumin ratio, blood urea nitrogen-to-creatinine ratio, and blood urea nitrogen-to-albumin ratio were used as inputs to a LR model (22). An XGB model was also shown to be highly effective when combined with blood test parameters such as HbA1c, total protein, hemoglobin, FPG, thyroid hormones, among others, as well as previous GDM and pre-pregnancy BMI (23).

LR and Random Forest (RF) models were implemented in one study, with the LR model showing better performance using 15 variables, including age, BMI, and blood test parameters (25). Both model types were also evaluated in another study; in this case, RF outperformed the LR model. The predictive variables included age, pre-pregnancy BMI, abdominal circumference in the first trimester, gravidity, polycystic ovary syndrome (PCOS), irregular menstruation, and family history of diabetes (29). In a study using data from an Iranian hospital, two hemoglobin measures, hematocrit, FBG, and red blood cell count were used as predictors (30). In another Iranian study, maternal age, BMI, FPG, and blood test parameters were analyzed for their predictive performance, with FPG identified as the most informative variable (34). Finally, an RF model using FPG and insulin-like growth factor binding protein 2 as inputs achieved the best performance among the evaluated ML models (33).

Age, pre-pregnancy BMI, PCOS, history of GDM, family history of GDM, FPG, and urine glucose were the variables used in an LR model in a study conducted in Western China (35). In another Chinese population study, age, BMI, HbA1c, and TH were used in an LR model (36). A Support Vector Machine (SVM) incorporating family history of diabetes, weight, white cell count, fasting glucose, and insulin was employed in another study, which also compared performance with other models such as LR, XGB, RF, and AdaBoost (37). A multilayer perceptron (MLP) using 11 metabolomics and 3 clinical features achieved the best predictive performance in a study in China (40).

Conventional predictors, such as age, pre-pregnancy BMI, family history of diabetes, plasma glucose and race/ethnicity, are used in combination with a wide variety of biomarkers to predict GDM, using two different time windows to make the prediction, 10–14 gestational weeks and 15–26 gestational weeks, with the best performance using all variables and with data available between 10–14 gestational weeks (39).

Electronic health records (EHRs) including maternal age, previous GDM, cholinesterase, FPG, HbA1c, sdLDL-C, thyroid stimulating hormone, APTT, and lymphocyte percentage were used to predict GDM using a LR model (24). EHR data from Israel comprising over 2000 features were analyzed using a Light Gradient Boosting Machine (LGBM), and an alternative model based on nine simple questions was also developed (26). XGB and LGBM were compared using maternal variables and laboratory data, with XGB outperforming LGBM. Stratification by nulliparity and multiparity was also evaluated. The authors also proposed a simplified questionnaire to facilitate clinical implementation based on feature selection methods (38).

In the state of the art, some studies use the IADPSG criteria (8) to diagnose GDM. The IADPSG criteria are based on findings from the Hyperglycemia and Adverse Pregnancy Outcome (HAPO) study (41). This one-step approach has been recommended by the World Health Organization since 2013 (42) and by the American Diabetes Association (ADA) since 2015 (4). Other diagnostic criteria are used in additional reports (17, 20, 26, 30, 38, 39).

### Deep learning

Deep learning (DL) has been applied across a wide range of fields, including healthcare, audio and speech processing, computer vision, translation, and sentiment analysis, among others (43). One of the most widely used network architectures is convolutional neural networks (CNNs) (43), which are inspired by the human visual system (44). In healthcare, CNNs have demonstrated the ability to identify complex, nonlinear patterns that can improve disease prediction and support clinical decision-making.

Patient variables obtained from routine clinical examinations or physical measurements, typically encoded as tabular data, remain challenging for DL models (45). To address this limitation, several approaches have proposed transforming tabular data into image representations, enabling the application of DL models originally designed for visual data (45). These approaches seek to exploit the superior pattern-recognition capability of CNNs while preserving the clinical information contained in routinely collected patient variables.

DeepInsight converts tabular data into images by arranging pixel locations such that features with similar characteristics are placed in close proximity (46). Bazgir et al. introduced a feature representation, termed REFINED, which generates a two-dimensional image based on pairwise feature relationships (47). Similarly, the image generator for tabular data (IGDT) positions features according to their mutual proximity (48). Sharma et al. proposed multiple strategies to map a one-dimensional feature vector into a two-dimensional graphical representation, including bar graphs, normalized distance matrices, and a hybrid of both approaches (49). MRep-DeepInsight, an extension of DeepInsight, proposes the use of multiple representations for each feature vector rather than the single representation used in the original framework (50). NCTD applies mathematical transformations, consisting of replication and row shifting, to simulate spatial relationships within the data, resulting in a two-dimensional synthetic image (51). Cubillos et al. proposed a transformation of tabular data into images by replicating features across rows and columns and improving the spatial arrangement via position permutations (52). All these methods exploit the representational power of CNNs for classification tasks and have demonstrated improved performance compared with traditional models in various application areas (46–52). However, their application to the early prediction of gestational diabetes mellitus has remained limited, despite the potential clinical value of identifying women at increased risk during early pregnancy. In addition, a multimodal framework combining MRI and tabular data has been employed to predict severe hemorrhage in placenta previa (53).

Motivated by the need for more accurate early prediction of GDM, we developed a deep learning framework designed to improve risk stratification using routinely collected at early pregnancy clinical data. By enabling more accurate identification of women at increased risk of GDM, the proposed approach has the potential to support earlier preventive interventions and clinical decision-making during pregnancy. The methodological innovations and principal contributions of this work are described in the following section.

### Our contributions

We developed a deep learning methodology for the early prediction of GDM by transforming routinely collected tabular clinical data into image representations, thereby enabling the application of CNN models. This approach leverages the ability of CNNs to identify complex nonlinear relationships among clinical variables while preserving the information contained in routinely collected patient data. The principal methodological innovation lies in optimizing the spatial arrangement of variables within the generated images using a genetic algorithm (GA), resulting in improved predictive performance compared with previous tabular-to-image transformation methods and with traditional machine learning approaches. The main contributions of this work are summarized as follows:

1. Development of a novel method for transforming tabular clinical data into images, enabling the use of CNN models that require 2D inputs. The method employs a GA to identify near-optimal positions for the clinical variables within the 2D representation.
2. Application of the proposed method to the early prediction of GDM, demonstrating improved predictive performance, particularly through substantial reductions in false-positive and false-negative classifications, thereby improving clinical risk stratification.
3. Comprehensive comparison of the proposed DL method with 12 machine learning models evaluated using the same dataset and reported in (54). as well as with the state-of-the-art tabular-to-image transformation method described in (52). The proposed approach demonstrated improvements across several performance metrics, including sensitivity, specificity, false-positive and false-negative rates, and area under the ROC curve.
4. The proposed method outperformed traditional ML models, achieving an average absolute specificity gain of 13.93 percentage points at matched sensitivity levels. In addition, it achieved an average absolute specificity gain of 9.62 percentage points compared with our previous deep learning approaches based on tabular-to-image transformation.
5. Integration of Grad-CAM explainability into the proposed framework, demonstrating that the model consistently attends to clinically relevant variables across patients, thereby supporting the validity and clinical interpretability of the tabular-to-image transformation approach for GDM prediction.

## Materials and methods

### Database

The database used in this study was obtained from the Obstetrics and Fetal Medicine Unit of the Hospital Parroquial de San Bernardo, Santiago, Chile, and includes data from 1,611 pregnancies between 2019 and 2022, including 229 cases of GDM, corresponding to an observed GDM prevalence of 14.21%. This hospital covers more than six municipalities with a population of more than 700,000. This is the same database used in our previous work (54). All patients included in the study had complete data for all variables. GDM diagnosis was based on the IADPSG/HAPO/WHO 2013/ADA criteria for gestational diabetes (4, 8, 41, 42), which include an OGTT (75 g), with fasting glycemia ≥ 92 mg/dL or 2-hour glycemia ≥ 153 mg/dL in the second trimester. Patients with preexisting Diabetes Mellitus were excluded. Data were collected during routine maternal visits up to the 20th week of gestation, with the first visit occurring after the 4th week of pregnancy. The dataset was partitioned at the patient level into independent training, validation, and test sets using stratified random sampling to preserve GDM prevalence across the three subsets. The same partition was used for all model comparisons and corresponds to that used in our previous work (54), thereby enabling direct comparison of model performance. The dataset was partitioned into three subsets: training set (1,127 patients), validation set (161 patients), and test set (323 patients, 39 positive cases). In the test set, the median first-visit gestational week was 11 (IQR: 9–13), with 77.7% of patients attending their initial visit during the first trimester (≤13 weeks) and 90.1% before week 16. The distribution of first-visit gestational age across the cohort is described in our previous work (54). The model can therefore issue predictions a median of 13 weeks before the standard OGTT performed at 24–28 weeks, providing a substantial lead time for preventive intervention for most patients. **Table 1** presents the variables collected in the dataset, organized by type: physical measurements, blood tests, previous pregnancies, substance use, and preexisting conditions or diseases. Although OGTT results were not used as predictive variables, they were included to provide the reference values and are presented as descriptive statistics. The OGTT protocol used at Hospital Parroquial de San Bernardo included fasting and 2-hour post-load glucose measurements; a 1-hour measurement was not obtained. GDM diagnosis was therefore based on fasting glucose ≥92 mg/dL and/or 2-hour glucose ≥153 mg/dL. Accordingly, the outcome definition represents a partial application of the IADPSG criteria because the 1-hour glucose criterion was not available.

**Table 1:** Clinical variables present in the dataset.

Variable/feature	Non-GDM women (1,382) mean (IQR)	GDM women (229) mean (IQR)
Age	27.64 (23-32)	31.11 (27-36)
Maternal Weight (First Control) [kg]	71.62 (60-81)	81.77 (69-92)
Height [m]	1.59 (1.55-1.63)	1.59 (1.55-1.63)
BMI (first control)	28.18 (24.03-31.64)	32.17 (28.16-35.83)
1TFG (First trimester fasting glycemia) [mg/dL]	77.22 (72-83)	87.12 (80-93)
OGTT (fasting) [mg/dL] *	74.28 (69-81)	95.48 (86-101)
OGTT (2hrs postprandial) [mg/dL] *	99.39 (84-114)	142.87 (120-171)
Gravidity	1.24 (0-2)	1.69 (0-2)
Parity	1.02 (0-2)	1.38 (0-2)
Abortions	0.22 (0-0)	0.32 (0-0)
Vaginal deliveries	0.79 (0-1)	1.03 (0-2)
Caesarean deliveries	0.22 (0-0)	0.34 (0-1)
Stillbirths	0.01 (0-0)	0.03 (0-0)
	(%)	(%)
Use of Tobacco	7.74	11.79
Use of Alcohol	3.62	4.80
Use of Illicit drugs	2.89	0.87
Cardiac Diseases	0.65	0.44
Biliary Diseases	1.01	2.18
Urinary Tract Disease	2.32	4.80
Chronic Kidney Diseases	0.36	0.00
Inflammatory Bowel Disease	0.07	0.44
Chronic Lung Diseases	2.31	3.05
Systemic lupus erythematosus/ Antiphospholipid antibody syndrome	0.14	0.44
Psychiatric Disorders	1.88	3.49
Endocrine Disorders	0.36	0.87
Gynecological Disorders	3.40	7.42
Epilepsy	1.09	0.44
Insulin Resistance	2.46	6.99
Hypothyroidism	4.05	9.17
Chronic Hypertension	4.70	12.66
Use of Antihypertensive Drugs	3.55	10.04

*OGTTs are not used in prediction.

### Data preprocessing

Continuous variables (Age, Maternal Weight, BMI, Parity, and first trimester fasting glycemia) were standardized using Z-score normalization. The mean and standard deviation were computed exclusively from the training set, and these statistics were applied to the validation and test sets to prevent information leakage across partitions. Binary variables (presence or absence of conditions such as chronic hypertension, hypothyroidism, insulin resistance, antihypertensive drug use, and other comorbidities) were encoded as 0/1 indicator values without further transformation.

### Data transformation

To match the 2D input format required by CNN models, patient data must be transformed into images. Typically, patient data are represented as a vector of length n (where n is the number of variables/features), commonly referred to as tabular data. This vector can be converted into a 2D image by replicating rows and columns, as proposed in (52), to meet CNN input requirements. The transformation process for continuous variables involves replicating the vector of patient variables to form a 2D matrix. Specifically, both rows and columns are replicated.

The tabular data for each participant, represented by five continuous variables, were transformed into a two-dimensional representation with a height of 250 and a width of 50 columns, matching the input dimensions used in our previous work (52). In the baseline transformation, variables are replicated across columns according to the predefined tabular-to-image mapping.

**Figure 1** illustrates this process, where n=5, representing avector with five variables, as shown in **Figure 1A**). In **Figure 1B**), thevector is replicated m times (15), resulting in a matrix withdimensions m×n. In **Figure 1C**), each column is replicated ktimes (3), producing the final matrix with dimensions m×nk (or15 x 15).

**Figure 1:**
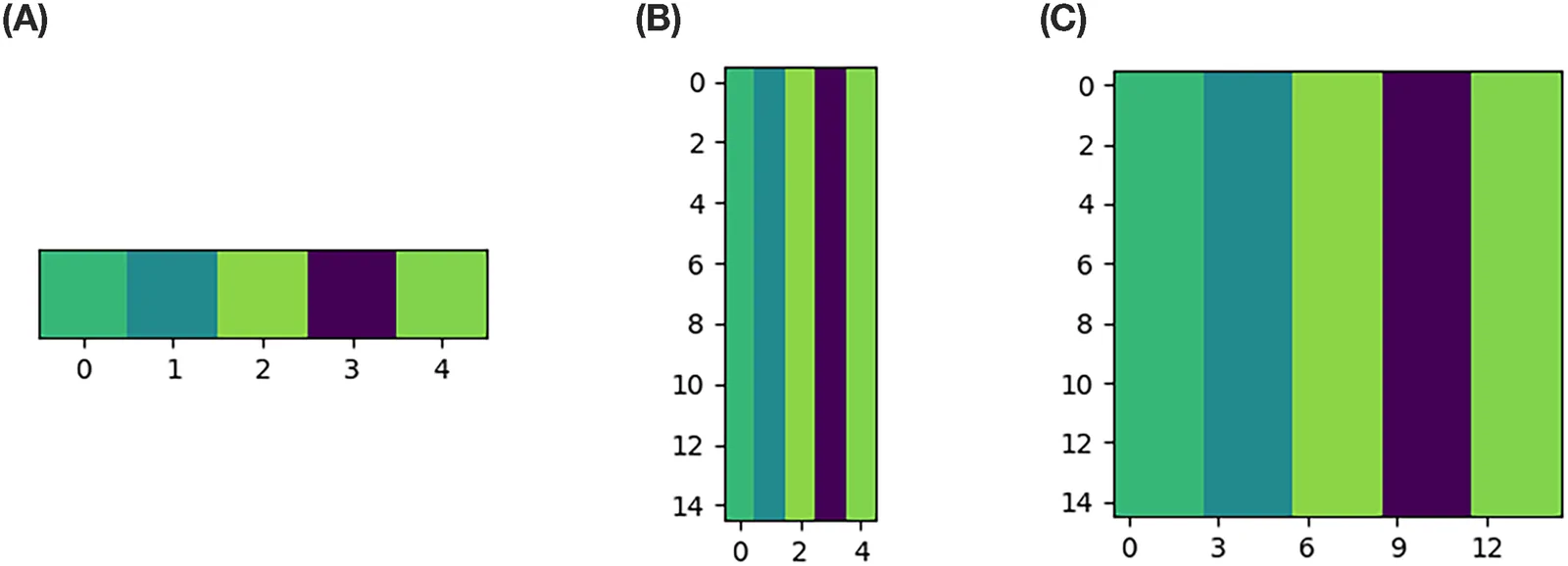
**(A)** Data from a patient with 5 variables represented as a vector (size: n). **(B)** Matrix obtained by replicating the vector m times, with m equal to 15 (size: m x n). **(C)** Matrix after column replication k times, with k equal to 3 (size: m x nk).

Binary and categorical variables are used as input to the classification stage, where the output of the CNN is concatenated with the binary variables into a new vector. This vector is then used as input to a MLP to produce the final prediction. **Figure 2** presents a block diagram illustrating how the transformation is applied to patient data in the case of seven continuous variables. In this study, n takes a maximum value of 7, corresponding to the seven continuous variables: age, maternal weight, height, BMI, gravidity, parity, and 1TFG. However, due to the similarity between gravidity and parity and the direct correlation of maternal weight and height with BMI, only five variables were used to construct the image. After preliminary testing, the selected values of m and k were 250 and 10, respectively, resulting in an image size of 250 × 50.

**Figure 2:**
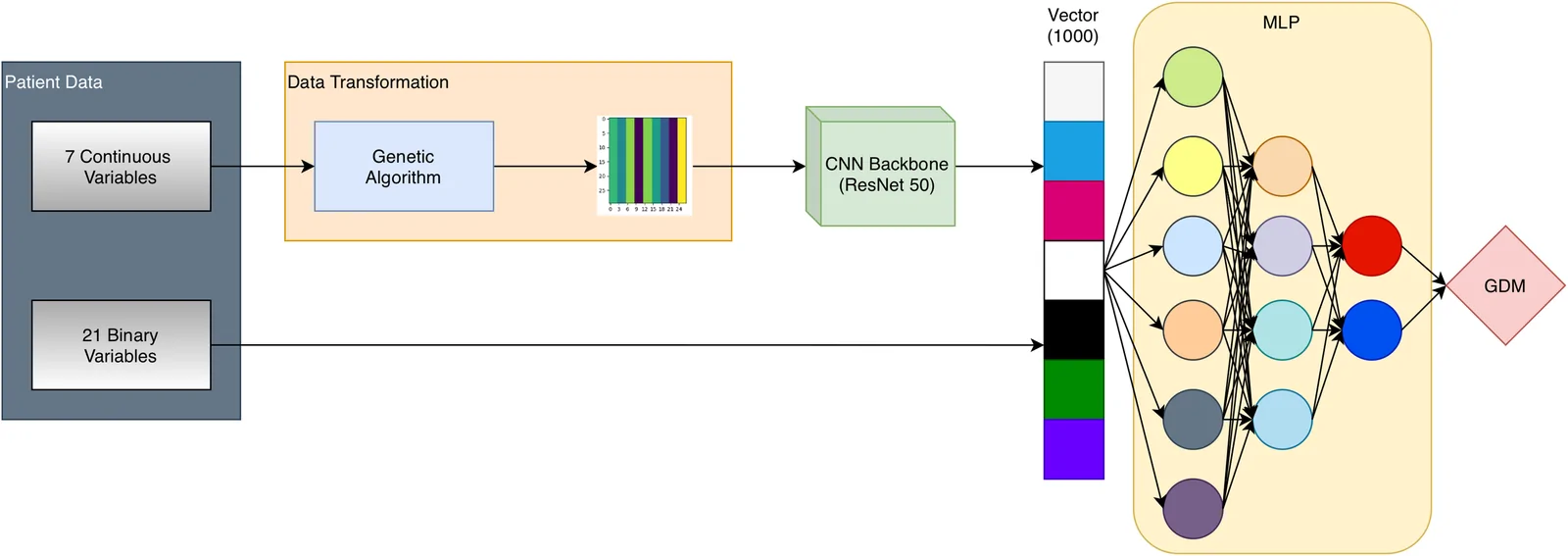
Block diagram of the proposed method, including input data, data transformation, and classification for GDM prediction.

The CNN selected for image feature extraction was ResNet-50 (55), pretrained on the ImageNet 2012 dataset (56). Pretrained weights (version 1.5) were implemented in PyTorch (57). The final fully connected (FC) classification layer of the CNN was removed, and the classification stage was replaced by an MLP comprising three hidden layers with 1000, 500, and 100 neurons, respectively, with dropout layers inserted between the hidden layers, following the architecture described in (52).

### Genetic algorithm for variable spatial arrangement

The spatial positions of the variables were evaluated as columns of size k (10) to determine the optimal arrangement, based on (52). Each variable was allowed to appear in more than one column. However, the computation of all possible combinations (5^50^) is computationally expensive, therefore, a genetic algorithm (GA) was used to search for a near-optimal arrangement of variable positions.

- Step 1: Each variable is encoded as a value, representing a gene in the genetic code.
- Step 2: The GA explores different positions for each variable, modifying the genotype. Given that the image width is 50 pixels, variables can be placed in multiple columns.
- Step 3: Each variable/column is replicated *m* times downward forming an image.
- Step 4: Each resulting image is evaluated using the validation set.

**Figure 3** illustrates a graphical representation of Steps 1 to 3 of the GA.

**Figure 3:**
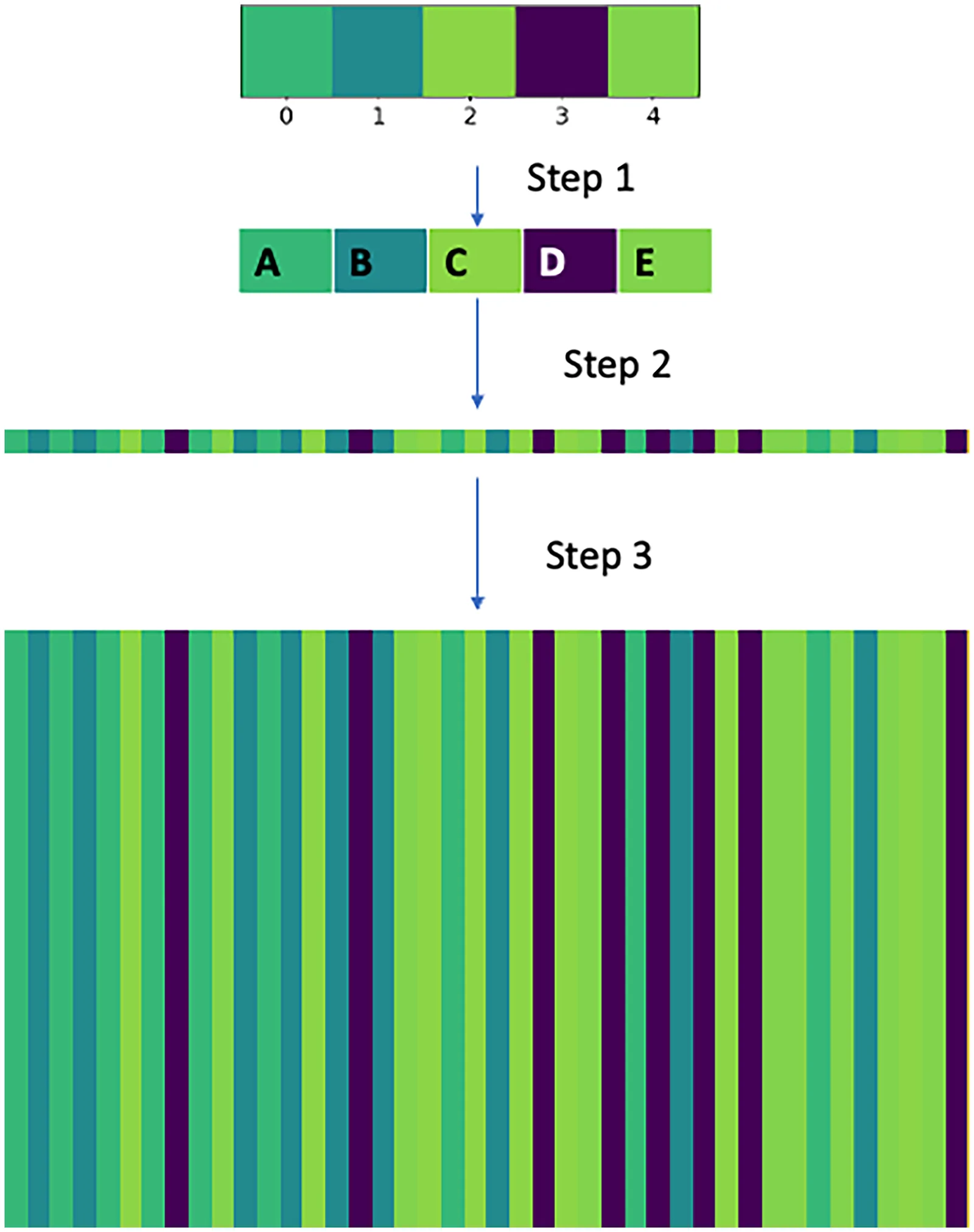
Example of the GA generating different images to represent patient data. Step 1: transform the patient variables into a code to be explored as a gene in the genotype. Step 2: the GA explores different solutions to find a near-optimal arrangement of the patient data. Step 3: each variable/column is replicated to generate an image.

For the GA-optimized representation, each of the 50 image columns was independently assigned one of the five continuous variables. Each candidate solution was therefore encoded as a length-50 vector of variable identifiers, yielding a search space of possible configurations. The fitness of each candidate configuration was evaluated by training the CNN using the resulting image representation and assessing its performance on the validation set.

To evaluate each image candidate, recall macro was used as the evaluation metric. The hyperparameters of the genetic algorithm were set as follows: Population size (40), elite individuals (10), number of generations (200), crossover probability (0.8), and mutation probability (0.02 and 0.2). The macro recall calculated by the algorithm represents the mean value obtained across five different seeds. The DL model resulting from our proposed method is referred to as DL Gen.

### Model interpretability via Grad-CAM

To assess the spatial decision patterns learned by the DL Gen model, Gradient-weighted Class Activation Mapping (Grad-CAM) (58) was applied to the test set. Grad-CAM generates class-discriminative heatmaps by combining feature maps from a target convolutional layer with the gradients of the predicted class score relative to those feature maps, thereby highlighting regions of the input that contribute most to the model prediction (58).

To translate spatial attention into variable-level importance, the genotype-encoded positions of each variable in the image was used to aggregate heatmap values. Because each variable occupied multiple columns in the genotype representation, variable importance was defined as the maximum heatmap activation across the columns assigned to that variable. Importance values were normalized to sum to one across all variables.

The statistical robustness of variable-level importance was assessed using a nonparametric bootstrap approach. For each group, 1,000 resampled datasets were generated by sampling patients with replacement, and variable importance was recalculated for each resample. The resulting distributions were used to derive 95% confidence intervals for each variable using the percentile method. The contrast between TP and TN importance, reflecting variables differentially used by the model to predict GDM versus non-GDM, was calculated as the paired bootstrap difference (TP - TN) to identify discriminative variables.

### Traditional machine learning models

We compared the performance of GDM prediction using our proposed method, DL Gen, with that of state-of-the-art ML models, all trained on the same database. The state-of-the-art ML models were implemented in our previous work (54). Twelve ML models were evaluated: Gaussian Naïve Bayes (GNB) and Bernoulli Naïve Bayes (BNB), Decision Trees (DT), SVMs, MLP, K-Nearest Neighbors (KNN), LR, RF, Extra Trees (ET) (59), Balanced Random Forest (BRF) (60), and Gradient Boosting (GB), using implementations of XGB (61) and LGBM (62). These models were trained using more than 3,000 combinations of hyperparameters to achieve optimal performance in GDM prediction.

### Data augmentation

Data Augmentation (DA) is a technique used in ML and DL to expand the size and diversity of training sets (63). DA usually improves model performance and reduces overfitting. DA consists primarily of modified versions of existing data. In our case, the newly generated data can be interpreted as additional patients in the training set. For this purpose, new data were generated within predefined boundaries. These boundaries aim to preserve medical plausibility in the artificially generated data. Only a subset of variables was modified to create DA within ranges relative to the original values. These ranges were defined by a specialist in Obstetrics/Gynecology and are referred to as the original range (OR). A limited range was also considered, as in (54). The variables modified were Age, Height, Weight, and 1TFG. As BMI is dependent on Height and Weight, it was adjusted according to variations in these variables. However, the generated data were required to remain within the same category based on the BMI classification recommended by the WHO (54, 64). Age was varied within ±2 years in the original range and ±1 year in the limited range. Height was altered by ±3 cm in the original range and ±1 cm in the limited range. Weight was modified by ±5 kg and ±2 kg in the original and limited ranges, respectively. Finally, 1TFG was changed within ±5 mg/dL and ±1 mg/dL for the respective ranges. Random rotations of ±10° and vertical flipping with a probability of 50% were also evaluated as DA for the generated images.

As images are used to represent variables in the proposed approach, DA alterations are applied by modifying the representation of each variable in the image. **Figure 4** shows an example of an image without DA (a) and the corresponding image with DA (b). Variables are modified by adding a random value within the predefined ranges. This modification changes each variable value by at most a given percentage, while remaining within the original range defined by the specialist. In this work, three DA strategies were considered: (i) generating equal numbers of negative and positive patients, (ii) generating only positive patients, and (iii) generating more positive than negative patients. The latter two strategies aim to balance the classes in the training dataset.

**Figure 4:**
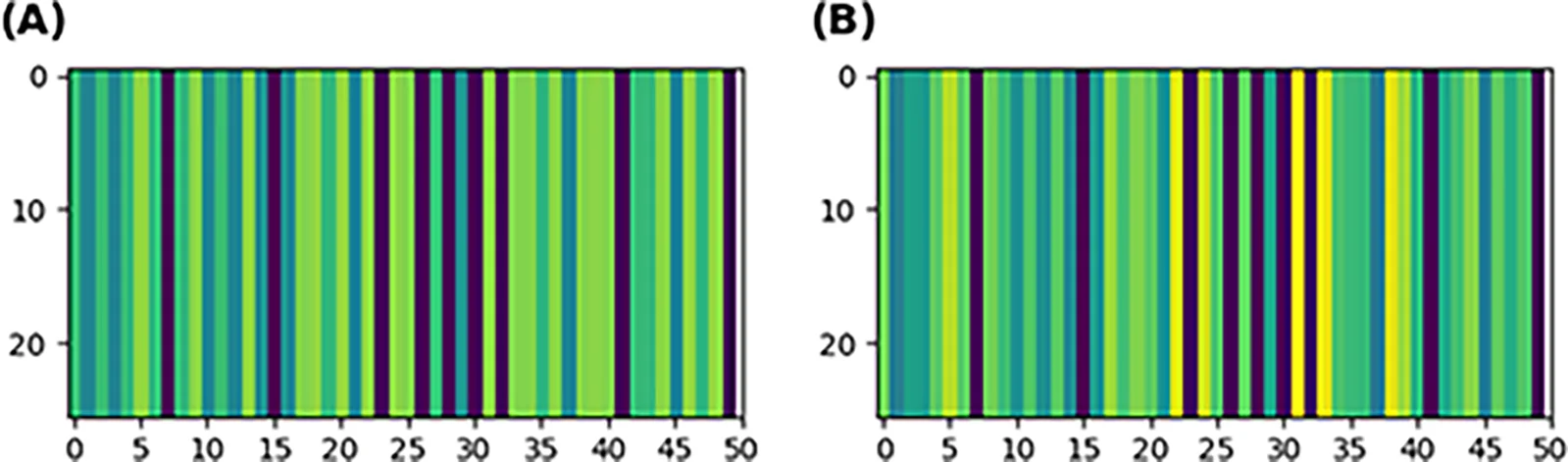
Example of DA. **(A)** Image without DA. **(B)** Image with DA, in which each column is altered by a different percentage.

### Model implementation and hyperparameters

The proposed models were implemented in Python 3.12.2 using the PyTorch framework (version 2.4.0) (57). Experiments were conducted on an NVIDIA GeForce RTX 4090 GPU. Traditional models were implemented in Python 3.9.12 using the Scikit-learn (59), Imbalanced-Learn (60), XGBoost (61), and LGBM (62) libraries (54). The hyperparameters used for the models are reported in **Table 2**.

**Table 2:** Hyperparameters used in each model, including those of the genetic algorithm.

Hyperparameter	Used by	Ranges [lower bound, upper bound]
*m*	DL Model	[1, 300]
*k*	DL Model	[1, 36]
Output Layer	DL Model	Last convolutional layer (a.k.a. ‘layer4.2.conv3’), Average Pooling layer (Avgpool)
CNN	DL Model	ResNet 50
Learning Rate	DL Model	0.0001
Optimizer	DL Model	Adam, AdamW
Loss Function	DL Model	Cross Entropy Loss
Dropout probability	DL Model	[0, 0.5]
Rotation	DL Model (DA)	No, ± 10°
Vertical flip probability	DL Model (DA)	No, 50%
Population	Genetic Algorithm	40
Elite	Genetic Algorithm	10
Generations	Genetic Algorithm	200
Crossover Probability	Genetic Algorithm	0.8
Mutation Probability	Genetic Algorithm	0.02, 0.2
“var_smoothing”	Gaussian Naïve Bayes	[1e-10, 1e-7]
“alpha”	Bernoulli Naïve Bayes	[1e-10, 1]
“criterion”	Decision Tree, Random Forest, Extra Trees, Balanced Random Forest	“gini”, “entropy”
“max_depth”	Decision Tree, Extreme Gradient Boosting	[1, 20]
“max_leaf_nodes”	Decision Tree	[6, 384]
“splitter”	Decision Tree	“best”, “random”
“kernel”	SVM	“linear”, “poly”, “rbf”, “sigmoid”
“degree”	SVM	[1, 3]
“C”	SVM, Logistic Regression	[0.0001, 10]
“solver”	Multi-Layer Perceptron	“sgd”, “adam”
“hidden_layer_sizes”	Multi-Layer Perceptron	[8, 256], hidden layers: [1, 10]
“activation”	Multi-Layer Perceptron	“logistic”, “tanh”, “relu”
“learning_rate_init”	Multi-Layer Perceptron	[0.001, 0.1]
“max_iter	Multi-Layer Perceptron	20000
“early_stopping”	Multi-Layer Perceptron	True, False
“learning_rate”	Multi-Layer Perceptron	“constant”, “invscaling”, “adaptive”
“algorithm”	K-Nearest Neighbors	“auto”, “ball_tree”, “kd_tree”, “brute”
“leaf_size”	K-Nearest Neighbors	[1, 30]
“p”	K-Nearest Neighbors	[1, 4]
“n_neighbors”	K-Nearest Neighbors	[1, 25]
“solver”	Logistic Regression	“newton-cg”, “lbfgs”, “liblinear”, “sag”, “saga”
“n_estimators”	Random Forest, Extra Trees, Balanced Random Forest, Extreme Gradient Boosting, Light Gradient Boosting Machine	[10, 2000]
“eta”	Extreme Gradient Boosting	[0.001, 0.3]
“booster”	Extreme Gradient Boosting	“gbtree”, “gblinear”, “dart”
“gamma”	Extreme Gradient Boosting	[0, 1]
“boosting”	Light Gradient Boosting Machine	“gbdt”, “rf”, “dart”, “goss”
“learning_rate”	Light Gradient Boosting Machine	[0.001, 0.1]

### Model evaluation and selection

The validation set was used to select the best models and to determine their decision thresholds. The test set was not used for training, model selection, or decision threshold determination. Instead, it was used to compute accuracy, sensitivity, specificity, macro-averaged recall, positive predictive value (PPV)/precision, area under the ROC curve (AUCROC), false positives (FP), false negatives (FN), and the total number of errors. As this is a screening task, high sensitivity/recall is prioritized to minimize FN. Therefore, sensitivities close to or above 0.80 (80%) were considered a key criterion in the analysis. For each target sensitivity level, the decision threshold for each model (DL Gen, DL without GA, and traditional ML) was determined exclusively from the validation set operating points. The threshold yielding the sensitivity closest to the target level was selected. When multiple thresholds yielded the same sensitivity, the threshold associated with the highest validation set macro averaged recall was selected. The resulting model specific thresholds were then fixed and applied unchanged to the independent test set, with no threshold selection or adjustment performed using the test set labels. On the test set, these validation derived thresholds yielded the same sensitivity across the three model types at each of the nine reported operating points. Because the test set contained 39 positive cases, equal sensitivity corresponds to identical TP and FN counts. However, the models differed in their classification of negative cases, as reflected by their different TN and FP counts and, consequently, by differences in specificity, accuracy, precision, and macro-averaged recall. Thus, **Table 3** compares the models at equivalent observed sensitivity levels, allowing their ability to reduce false positives and improve specificity while maintaining the same level of positive case detection to be assessed directly.

**Table 3:** Results of the proposed model (DL Gen) compared with those obtained from traditional ML models and DL models without GA, included for comparison, for sensitivities ≥ 0.7949.

Model number	Model type	Variables	Image size	Accuracy	Sensitivity	Specificity	Recall macro	PPV	AUC ROC	TN	FP	FN	TP	FP+FN
**1**	**DL Gen**	**5**	**250x50**	**0.6037**	**1**	**0.5493**	**0.7746**	**0.2335**	**0.8414**	**156**	**128**	**0**	**39**	**128**
D1	DL	5+10B	250x50	0.4118	1	0.3310	0.6655	0.1703	0.7885	94	190	0	39	190
T1	MLP	12	–	0.3993	1	0.3169	0.6584	0.1674	0.8188	90	194	0	39	194
**2**	**DL Gen**	**5**	**250x50**	**0.6316**	**0.9744**	**0.5845**	**0.7794**	**0.2436**	**0.8329**	**166**	**118**	**1**	**38**	**119**
D2	DL	5+10B	250x50	0.6192	0.9744	0.5704	0.7724	0.2375	0.7853	162	122	1	38	123
T2	MLP	8	–	0.5511	0.9744	0.4930	0.7337	0.2088	0.8002	140	144	1	38	145
**3**	**DL Gen**	**5**	**250x50**	**0.6718**	**0.9487**	**0.6338**	**0.7913**	**0.2624**	**0.8318**	**180**	**104**	**2**	**37**	**106**
D3	DL	5	250x50	0.6316	0.9487	0.5880	0.7684	0.2403	0.8096	167	117	2	37	119
T3	SVM	5	–	0.6068	0.9487	0.5598	0.7542	0.2284	0.8234	159	125	2	37	127
**4**	**DL Gen**	**5**	**250x50**	**0.7864**	**0.9231**	**0.7676**	**0.8453**	**0.3529**	**0.8519**	**218**	**66**	**3**	**36**	**69**
D4	DL	5+10B	250x50	0.6471	0.9231	0.6092	0.7661	0.2449	0.7853	173	111	3	36	114
T4	SVM	5	–	0.6130	0.9231	0.5704	0.7468	0.2278	0.8234	162	122	3	36	125
**5**	**DL Gen**	**5**	**250x50**	**0.7895**	**0.8974**	**0.7746**	**0.8360**	**0.3535**	**0.8422**	**220**	**64**	**4**	**35**	**68**
D5	DL	5	250x50	0.6873	0.8974	0.6585	0.7779	0.2652	0.8108	187	97	4	35	101
T5	MLP	5	–	0.6594	0.8974	0.6268	0.7621	0.2482	0.8199	178	106	4	35	110
**6**	**DL Gen**	**5**	**250x50**	**0.8050**	**0.8718**	**0.7958**	**0.8338**	**0.3696**	**0.8470**	**226**	**58**	**5**	**34**	**63**
D6	DL	5	250x50	0.7183	0.8718	0.6972	0.7845	0.2833	0.8093	198	86	5	34	91
T6	MLP	7	–	0.6873	0.8718	0.6620	0.7669	0.2615	0.8160	188	96	5	34	101
**7**	**DL Gen**	**5+8B**	**250x50**	**0.8235**	**0.8462**	**0.8204**	**0.8333**	**0.3929**	**0.8425**	**233**	**51**	**6**	**33**	**57**
D7	DL	5	250x50	0.7709	0.8462	0.7606	0.8034	0.3267	0.8033	216	68	6	33	74
T7	MLP	6	–	0.7090	0.8462	0.6901	0.7681	0.2727	0.8142	196	88	6	33	94
**8**	**DL Gen**	**5**	**250x50**	**0.8607**	**0.8205**	**0.8662**	**0.8434**	**0.4571**	**0.8331**	**246**	**38**	**7**	**32**	**45**
D8	DL	5+10B	250x50	0.7771	0.8205	0.7711	0.7958	0.3299	0.8183	219	65	7	32	72
T8	SVM	12	–	0.7554	0.8205	0.7465	0.7835	0.3077	0.8135	212	72	7	32	79
**9**	**DL Gen**	**5**	**250x50**	**0.8514**	**0.7949**	**0.8592**	**0.8270**	**0.4366**	**0.8164**	**244**	**40**	**8**	**31**	**48**
D9	DL	5+10B	250x50	0.7988	0.7949	0.7993	0.7971	0.3522	0.8232	227	57	8	31	65
T9	SVM	7	–	0.7399	0.7949	0.7324	0.7636	0.2897	0.8143	208	76	8	31	84

B: Binary variables. Variables: Number of Variable.

B: Binary variables. Variables: Number of Variables.

For each model, performance metrics including accuracy, sensitivity, specificity, macro recall, positive predictive value (PPV)/precision, and AUC-ROC are reported, together with the confusion matrix values (TP, TN, FP, and FN). The best results at each sensitivity level are highlighted in bold.

For each trained model configuration, decision thresholds were determined exclusively on the validation set to achieve target sensitivity levels ranging from 0.79 to 1.00. Among the models evaluated at each target sensitivity level, the best-performing configuration was selected based on validation-set macro-averaged recall. Once this selection procedure was completed, the selected model configurations and their corresponding validation-derived decision thresholds were fixed and subsequently applied unchanged to the independent test set. No test-set labels or performance metrics were used for model selection, threshold determination, threshold adjustment, or model retuning. Therefore, the test-set sensitivities and TP counts reported in **Tables 3**–**5** are outcomes obtained after applying the fixed validation-derived thresholds and were not target operating points selected on the test set. After completion of the independent test-set evaluation, **Table 3** was ordered by the observed test-set sensitivity to facilitate comparison across operating points and allow readers and clinicians to identify models with different sensitivity–specificity trade-offs. This post-evaluation ordering had no role in model selection or decision-threshold determination.

**Table 4:** Summary of the improvements achieved by the proposed DL Gen model in specificity, number of true negatives (TN), and total errors (FP+FN) at different sensitivity levels compared with traditional ML models.

Sensitivity	Relative specificity improvement (%)	Absolute specificity improvement (percentage points)	Change in TN	Change in total errors (FP+FN)
1.0000	73.34	+23.24	90 → 156	194 → 128
0.9744	18.56	+9.15	140 → 166	145 → 119
0.9487	13.22	+7.39	159 → 180	127 → 106
0.9231	34.57	+19.72	162 → 218	125 → 69
0.8974	23.58	+14.79	178 → 220	110 → 68
0.8718	20.21	+13.38	188 → 226	101 → 63
0.8462	18.88	+13.03	196 → 233	94 → 57
0.8205	16.03	+11.97	212 → 246	79 → 45
0.7949	17.31	+12.68	208 → 244	84 → 48

Relative specificity improvement indicates the percentage increase in specificity achieved by DL Gen compared with traditional ML, whereas absolute specificity improvement indicates the corresponding difference in percentage points. Across the nine sensitivity levels, the mean and median relative specificity improvements were 26.19% and 18.88%, respectively, and the mean absolute specificity improvement was 13.93 percentage points. DL Gen achieved, on average, 39.56 additional true negatives and 39.56 fewer total classification errors at matched sensitivity levels.

**Table 5:** Summary of the improvements achieved by the proposed DL Gen model in specificity, number of true negatives (TN), and total errors (FP+FN) at different sensitivity levels compared with DL models without GA.

Sensitivity	Relative specificity improvement (%)	Absolute specificity improvement (percentage points)	Change in TN	Change in total errors (FP+FN)
1.0000	65.95	+21.83	94 → 156	190 → 128
0.9744	2.47	+1.41	162 → 166	123 → 119
0.9487	7.79	+4.58	167 → 180	119 → 106
0.9231	26.00	+15.85	173 → 218	114 → 69
0.8974	17.63	+11.62	187 → 220	101 → 68
0.8718	14.14	+9.86	198 → 226	91 → 63
0.8462	7.86	+5.99	216 → 233	74 → 57
0.8205	12.33	+9.51	219 → 246	72 → 45
0.7949	7.49	+5.99	227 → 244	65 → 48

Relative specificity improvement indicates the percentage increase in specificity achieved by DL Gen compared with DL without GA, whereas absolute specificity improvement indicates the corresponding difference in percentage points. Across the nine sensitivity levels, the mean and median relative specificity improvements were 17.96% and 12.33%, respectively, and the mean absolute specificity improvement was 9.62 percentage points. DL Gen achieved, on average, 27.33 additional true negatives and 27.33 fewer total classification errors at matched sensitivity levels.

Bootstrap 95% confidence intervals based on 1,000 resamples were computed for sensitivity, specificity, PPV, and AUC-ROC for all models reported. In addition, paired bootstrap analyses based on 1,000 resamples were performed to compare specificity and PPV between DL Gen and the corresponding DL without GA and traditional ML models at each matched sensitivity level. Because the compared models were evaluated on the same test-set participants, predictions were resampled in a paired manner to preserve the within-participant dependence between models. For each comparison, the difference in specificity or PPV, its 95% confidence interval, and a two-sided p-value.

### Calibration

To complement the discrimination metrics, calibration of the recommended model (DL Gen 8) and the two baseline models at the corresponding operating point was assessed using the Brier score, calibration slope and intercept, Expected Calibration Error (ECE), and reliability diagrams based on 10 equal-frequency bins. Decision Curve Analysis (DCA) (65) was additionally performed to compare the net clinical benefit of DL Gen 8 with the DL 8 and traditional SVM baselines, as well as with the Treat All and Treat None strategies, across threshold probabilities from 0 to 40%. Both analyses were performed using test-set predictions.

## Results

### Genetic results

The GA was used to determine the spatial locations of patients’ continuous variables during the transformation of tabular data into images. Five different seeds were tested to initialize the GA population. The evolution of the best-performing GA is shown in **Figure 5**. This figure presents fitness as a function of generation number for the best individual (blue), the mean fitness of the elite (orange), and the mean fitness of the entire population (green). The evolution obtained with the other seed initializations is shown in **Supplementary Figures S1**–S4 of **Supplementary Material**.

**Figure 5:**
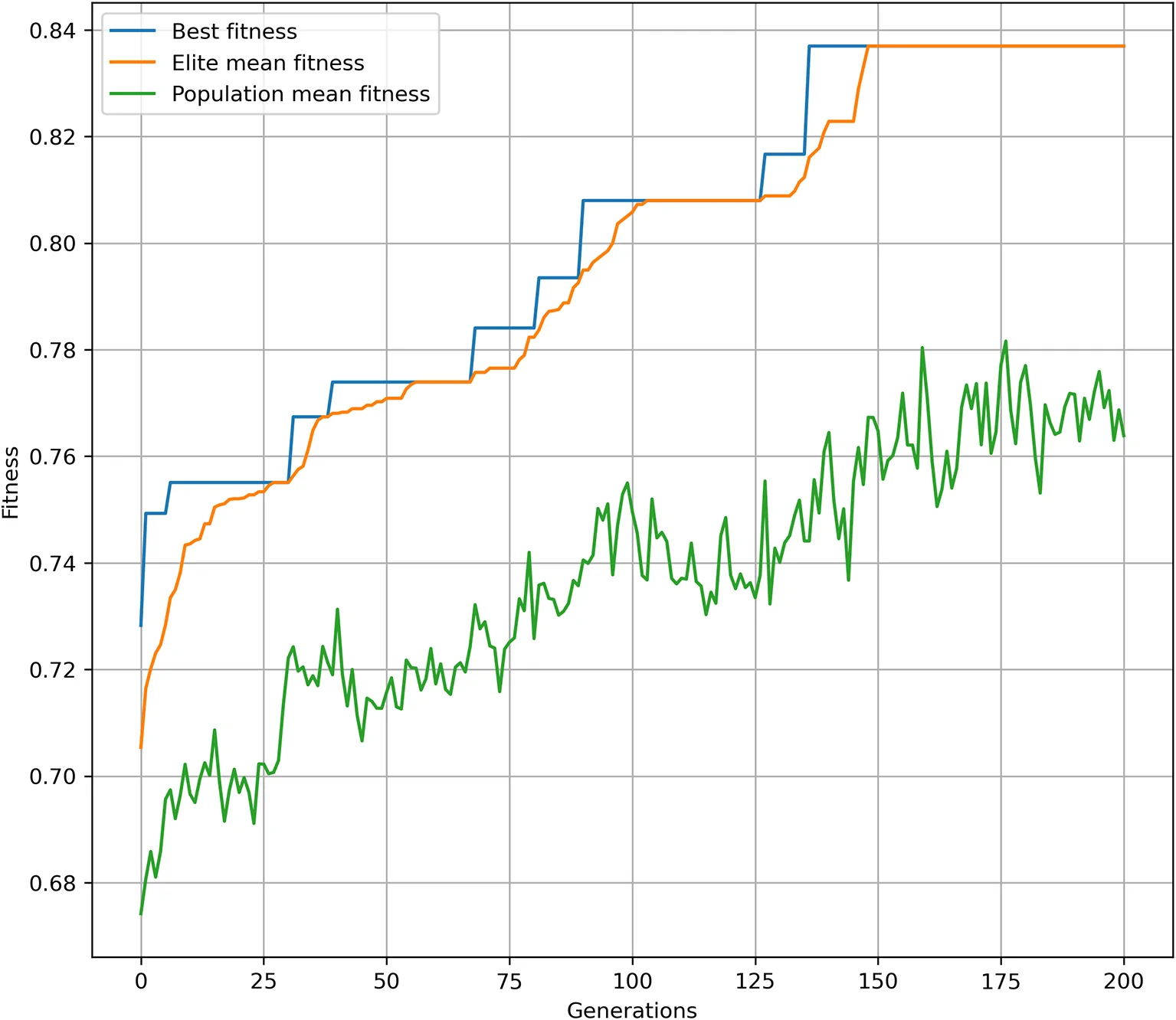
Fitness evolution for the best seed. Blue denotes the best fitness, orange the mean fitness of the elite (10 individuals), and green the mean fitness of the population.

### Model performance

The results of the proposed DL Gen model are reported in **Table 3**, together with those obtained from traditional ML models and DL models without GA for comparison. **Table 3** summarizes the number of variables used, the image size for the DL Gen and DL models. For each model, performance metrics including accuracy, sensitivity, specificity, macro recall, positive predictive values (PPV)/precision, and AUC-ROC are reported, together with the numbers of false positives (FP), false negatives (FN), true positives (TP), true negatives (TN), and the total number of errors (FP+FN). The FP, FN, TP, and TN values constitute the confusion matrix for each model. The spatial positions of the variables for each model, as well as the output layer, binary variables and optimizer used for the DL Gen and DL models, are provided in **Supplementary Table S1** of the **Supplementary Material**. **Supplementary Table S2** of **Supplementary Material** shows DA configuration used. All models were evaluated using an independent test set partition not used in any previous steps of the method. As the proposed approach is applied to a GDM screening process, the analysis focuses on the high-sensitivity range. As an additional comparison, we included the results of gradient boosting baselines (XGBoost and LightGBM) calculated in our previous work but not presented before (54).

**Table 4** summarizes the improvements achieved by the DL Gen model in specificity, the number of TN, and the total number of errors (FP+FN) at each sensitivity level, compared with traditional ML models. **Table 5** presents the same summary in comparison with DL models without GA.

Results of traditional ML models without optimization (variable selection, DA, and hyperparameter tuning) are presented in **Supplementary Table S4** of the **Supplementary Material**.

**Figure 6** shows the total number of errors (FP+FN) as a function of detected TP, i.e., total errors at different sensitivity levels. The proposed DL Gen model is represented by the green line; the DL model without GA by the orange line; traditional ML models by the blue line; and traditional ML models without optimization by the red curve. The maximum sensitivity of 1 is reached at 39 TP.

**Figure 6:**
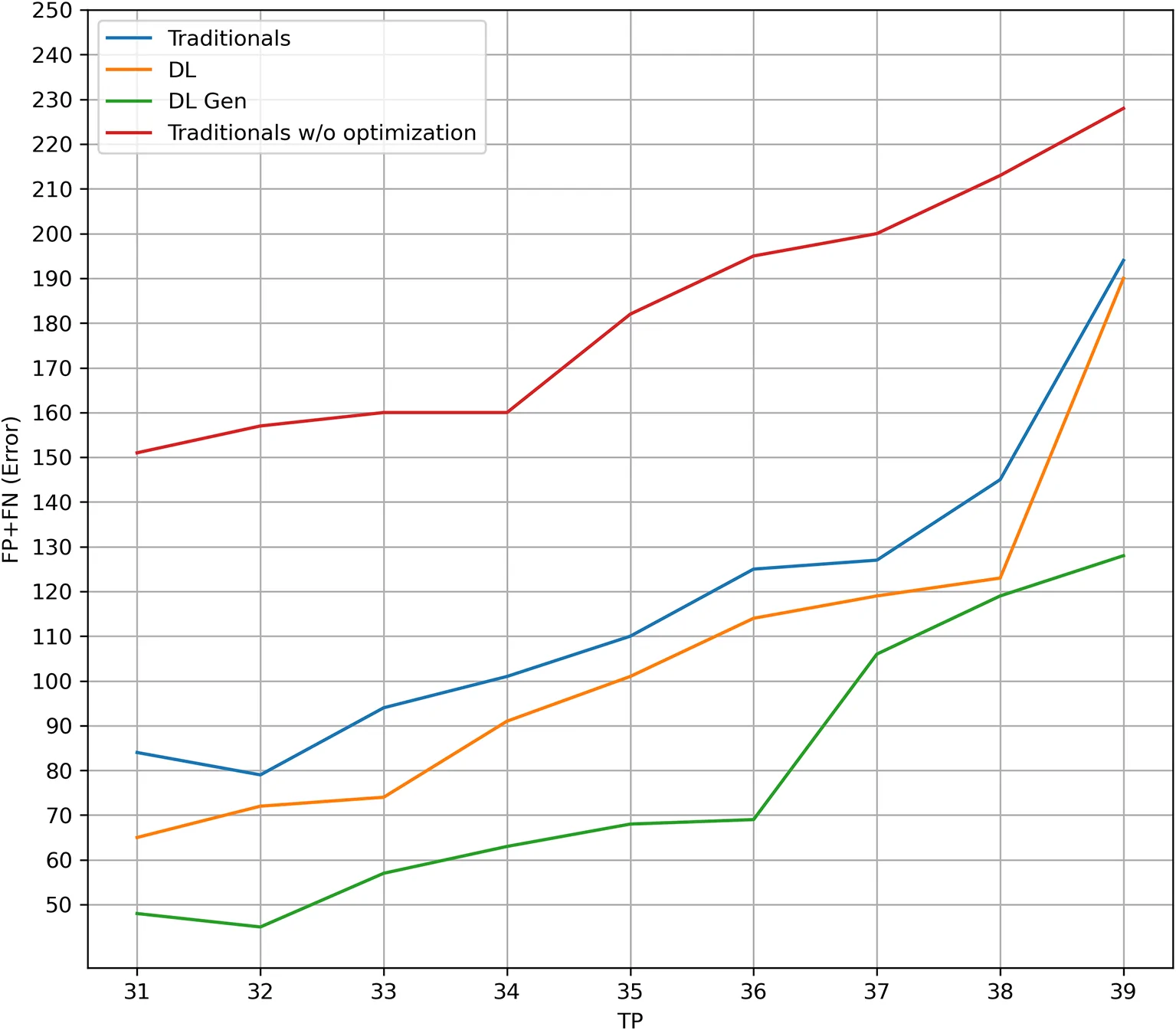
Errors (FP+FN) based on true positives detected by the models in the test set.

**Figures 7**, **8** show ROC curves for DL Gen models (3 and 7), DL models without GA (D3 and D7), traditional ML models without GA (T3 and T7) and ML models without optimization (MLP, 31 variables, 100 hidden neurons).

**Figure 7:**
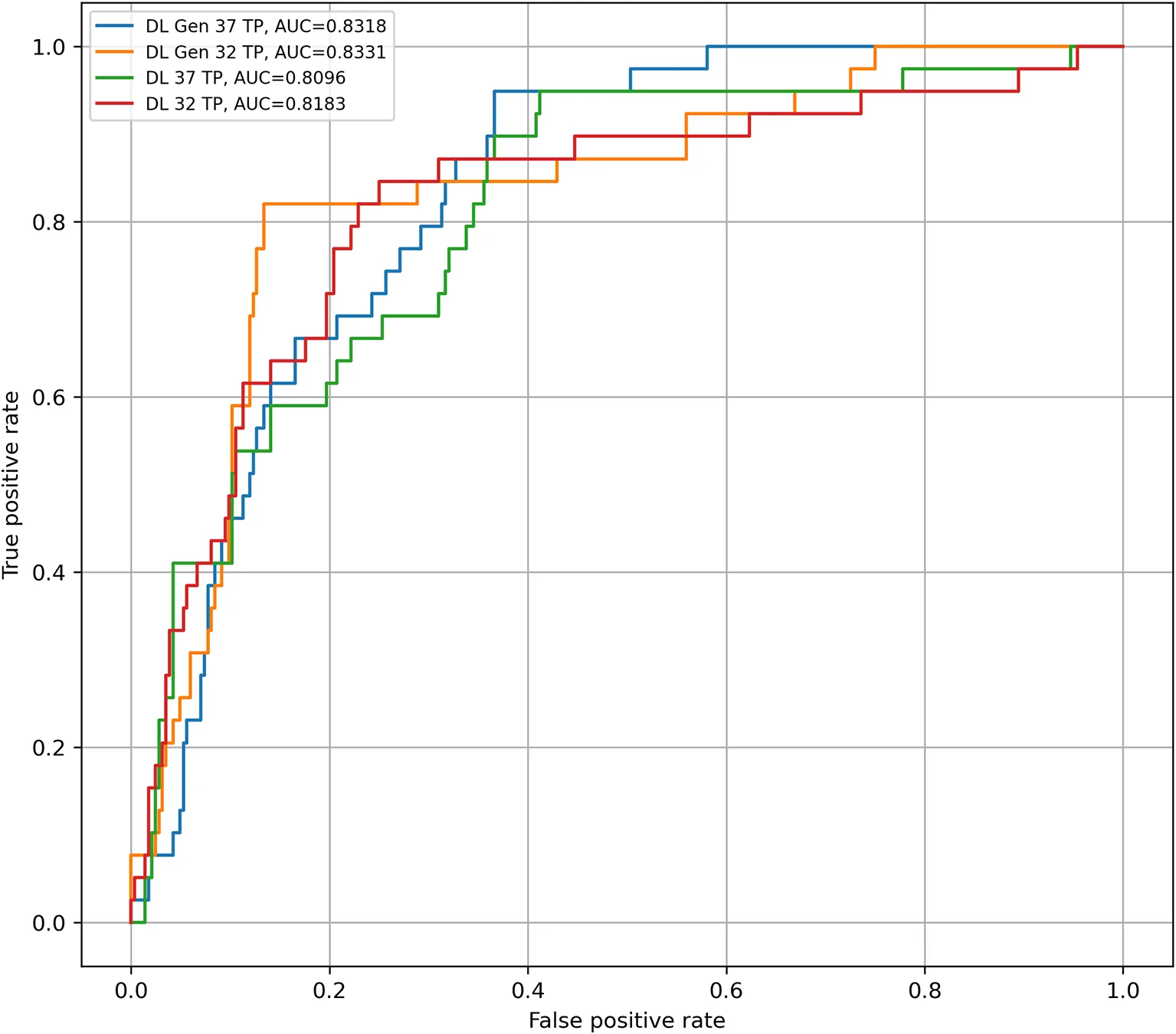
ROC curves of the DL Gen models (3 and 8) compared with DL models without GA (D3 and D7), at TP values of 32 and 37.

**Figure 8:**
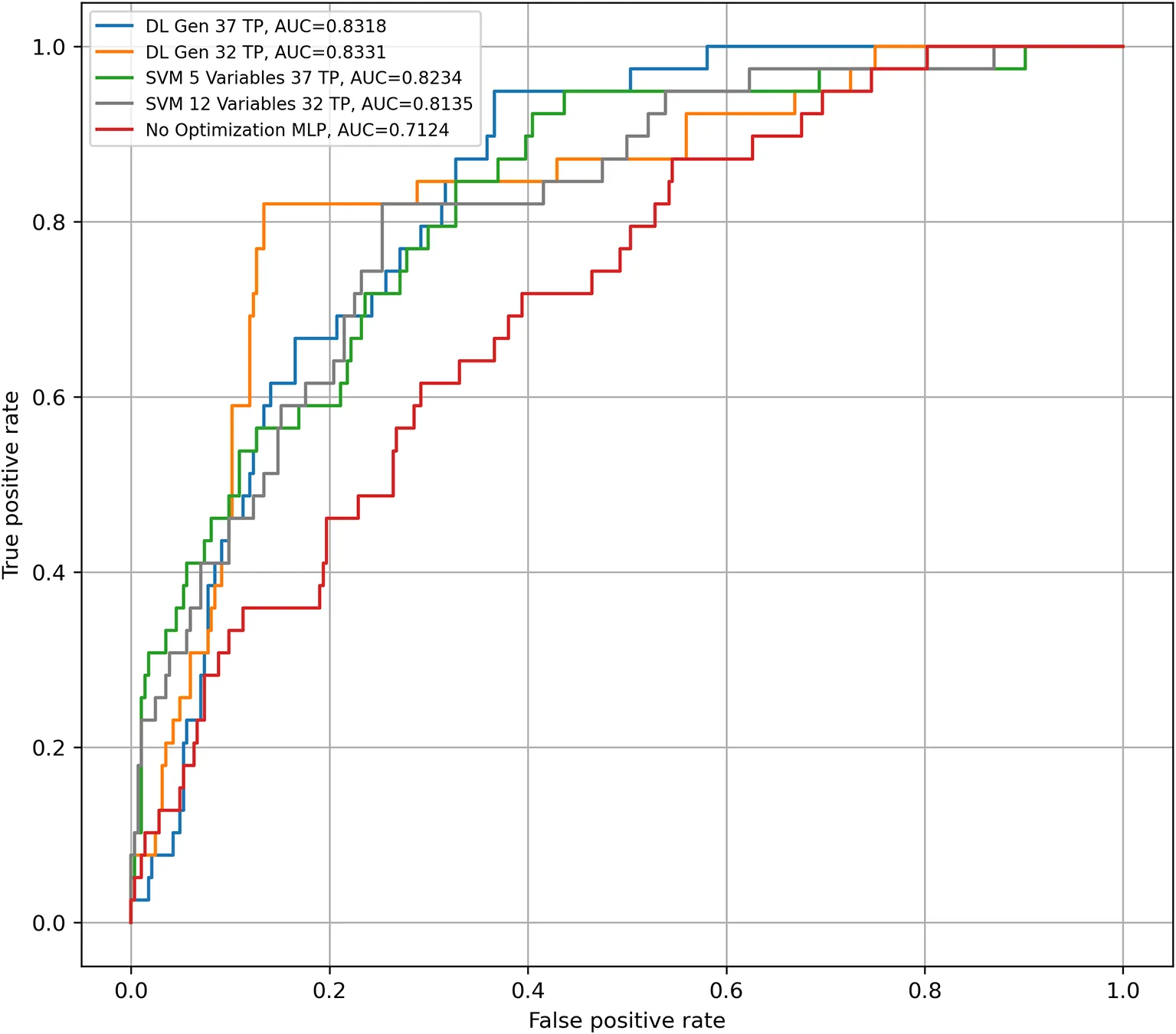
ROC curves of the DL Gen models (3 and 8) compared with the traditional ML models (SVMs, T3 and T7) from our previous work (54) on GDM and an MLP without optimization (NO1), at TP values of 32 and 37.

The parameters m and k were set to 250 and 10, respectively, after testing various combinations (see **Supplementary Material**, **Supplementary Tables S5**, **S6**).

In **Supplementary Material**, **Supplementary Table S7** presents a comparison between DL Gen models with DA versus No DA for the analyzed range of sensitivities.

Calibration analysis showed similar Brier scores (0.088–0.091) and low ECE values (≤0.06) across the three models. For DL Gen 8, the calibration slope (1.44) and intercept (0.72) indicated some deviation from ideal calibration. However, the reliability diagram showed good agreement between predicted and observed outcomes in the lower-probability range (0.00–0.20), where most predictions in the test set were concentrated (**Supplementary Figure S5**). DCA showed a higher net benefit for DL Gen 8 than for both baseline models across the clinically relevant threshold range of 5–20% (**Supplementary Figure S6**).

Validation and test set performance of the nine DL Gen models is compared in **Supplementary Table S8**. Defining the performance gap as validation minus test performance, the mean signed gap across the nine models was −0.048 for AUC-ROC and −0.015 for macro-averaged recall. Test set AUC-ROC exceeded validation-set AUC-ROC for all nine models, while test-set macro-averaged recall exceeded validation performance for six of the nine models. Thus, the observed validation-to-test gaps did not indicate systematic validation-set optimism.

Bootstrap analysis was used to quantify uncertainty in test-set performance, with 95% confidence intervals calculated for sensitivity, specificity, PPV, and AUC-ROC (**Supplementary Table S9**). At the recommended operating point (Model 8), DL Gen achieved sensitivity 0.821 [95% CI: 0.692, 0.929], specificity 0.866 [95% CI: 0.825, 0.905], PPV 0.457 [95% CI: 0.339, 0.580], and AUC-ROC 0.833 [95% CI: 0.760, 0.899]. Paired bootstrap comparisons between DL Gen and the baseline models at matched sensitivity levels showed significantly higher specificity and PPV for DL Gen than traditional ML at all nine operating points. Compared with DL without GA, DL Gen showed significantly higher specificity at 7 of 9 operating points and significantly higher PPV at 6 of 9 operating points (**Supplementary Table S10**). At the recommended operating point, DL Gen increased specificity by 0.095 [95% CI: 0.052, 0.142] and PPV by 0.127 [95% CI: 0.054, 0.206] relative to DL without GA, and increased specificity by 0.120 [95% CI: 0.072, 0.171] and PPV by 0.149 [95% CI: 0.077, 0.229] relative to traditional ML. All four paired comparisons were statistically significant.

Participants with early-pregnancy 1TFG ≥92 mg/dL represented 8.7% (140/1,611) of the original cohort and were similarly distributed across the training (8.8%), validation (8.1%), and test (8.7%) partitions (**Supplementary Table S11**). In the original independent test set, the criterion 1TFG ≥92 mg/dL alone identified 14 of 39 GDM cases (sensitivity = 0.359), whereas the recommended DL Gen model identified 33 of 39 GDM cases, including 19 cases with 1TFG <92 mg/dL.

To further assess the potential influence of the overlapping diagnostic criterion, all participants with early-pregnancy 1TFG ≥92 mg/dL were excluded from the training, validation, and test partitions before model retraining and evaluation. The resulting filtered partitions comprised 1,028 training participants (118 GDM), 148 validation participants (17 GDM), and 295 test participants (25 GDM). Three representative DL Gen models spanning the clinically relevant sensitivity range (Models 4, 6, and 8) were retrained on the filtered training data, with hyperparameter selection and decision-threshold determination performed using the filtered validation set. The filtered test set was reserved exclusively for final evaluation, without further model or threshold adjustment.

On the filtered independent test set, Model 4 achieved an AUC-ROC of 0.755 (sensitivity = 0.920; specificity = 0.633), Model 6 an AUC-ROC of 0.790 (sensitivity = 0.880; specificity = 0.741), and the recommended Model 8 an AUC-ROC of 0.810 (sensitivity = 0.800; specificity = 0.796). The corresponding AUC-ROC values in the original analysis were 0.852, 0.847, and 0.833, respectively. Thus, after complete exclusion of participants with early-pregnancy 1TFG ≥92 mg/dL from all data partitions and subsequent model retraining, the models retained discriminatory performance, with the recommended Model 8 showing an AUC-ROC difference of −0.023. These findings indicate that the predictive performance of DL Gen does not depend on the inclusion of participants with early-pregnancy 1TFG ≥92 mg/dL (**Supplementary Tables S11**, **S12**).

### Interpretability analysis

Test set patients (n=323) were classified into four groups based on model 8: true positives (TP, n=32), true negatives (TN, n=246), false positives (FP, n=38), and false negatives (FN, n=7). For each group, individual Grad-CAM heatmaps were averaged to obtain a representative spatial attention pattern, and standard deviations were computed per pixel as a measure of inter-patient consistency. Grad-CAM was applied to the same DL Gen model used in the performance analysis (Model 8, **Table 3**), ensuring consistency between performance and interpretability findings.

Grad-CAM analysis revealed that the DL Gen model learned two spatially distinct attention patterns associated with positive and negative predictions. **Figure 9** shows the average Grad-CAM heatmap, the corresponding pixel-wise standard deviation, and variable-level importance ranking for each of the four prediction groups (TP, TN, FP, FN). Heatmaps for patients predicted as GDM (TP and FP) consistently exhibited focused activation in the left region of the input image (approximately columns 0–15 of the genotype), whereas heatmaps for patients predicted as non-GDM (TN and FN) showed activation concentrated in the right region (approximately columns 35–48). Standard deviations across patients within each group remained low (maximum values < 0.045 on a [0,1] scale), indicating that these spatial patterns were highly consistent across individuals. The mapping between genotype positions and clinical variables is provided in **Supplementary Material** (**Supplementary Figure S7**).

**Figure 9:**
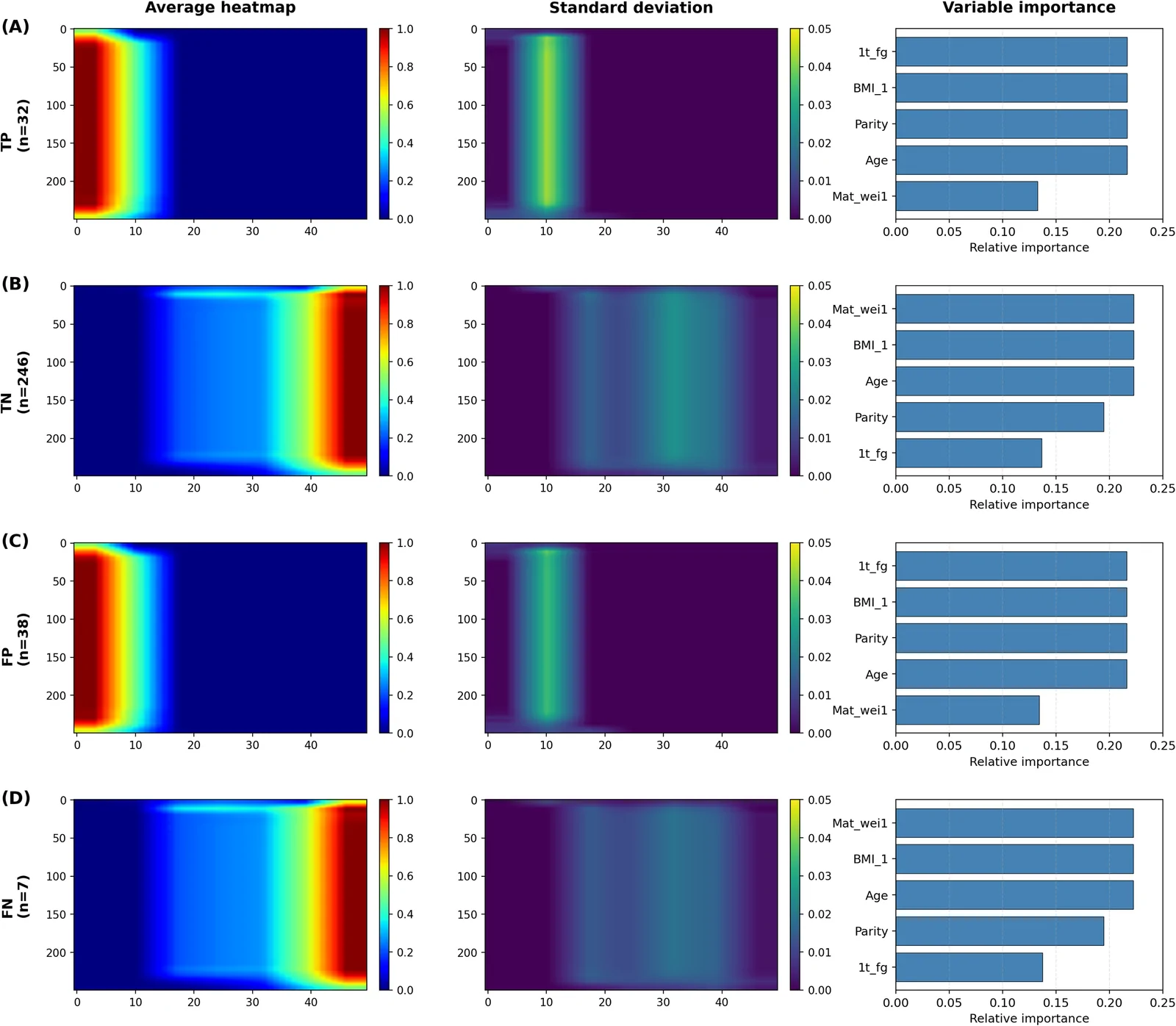
Grad-CAM analysis by prediction group on the test set (n=323) using DL Gen Model 8. Rows correspond to true positives (TP, n=32), true negatives (TN, n=246), false positives (FP, n=38), and false negatives (FN, n=7). For each group: (left) average Grad-CAM heatmap across patients, normalized to [0,1]; (center) per-pixel standard deviation of the heatmap, indicating the consistency of the spatial attention pattern across patients; and (right) variable-level importance ranking obtained by aggregating heatmap activations across the genotype positions assigned to each variable (maximum aggregation, normalized to sum to one across variables).

The genotype-based aggregation of heatmap activations converts these spatial patterns into variable-level importance on **Figure 10**. Bootstrap analysis (1,000 iterations) of the difference in variable importance between TP and TN groups identified three variables with substantially different attention allocation across prediction directions, all consistent with established clinical risk factors for GDM. First trimester fasting glycemia (1TFG) and Parity were preferentially focused when predicting GDM (TP–TN: +0.080, 95% CI [+0.080, +0.081], and +0.022, 95% CI [+0.022, +0.023], respectively), whereas Maternal Weight was preferentially attended when predicting non-GDM (TP–TN: -0.090, 95% CI [-0.092, -0.088]). Age and BMI showed near-zero TP–TN differences (|TP–TN| < 0.01). Although these contrasts were technically distinguishable from zero given the narrow bootstrap confidence intervals, their magnitudes were approximately 15-fold smaller than those of the leading discriminators, supporting their interpretation as background-context variables rather than decision-driving features. The narrow confidence intervals reflect the deterministic and consistent attention patterns the model applies across patients within each group, rather than the magnitude of population-level effects.

**Figure 10:**
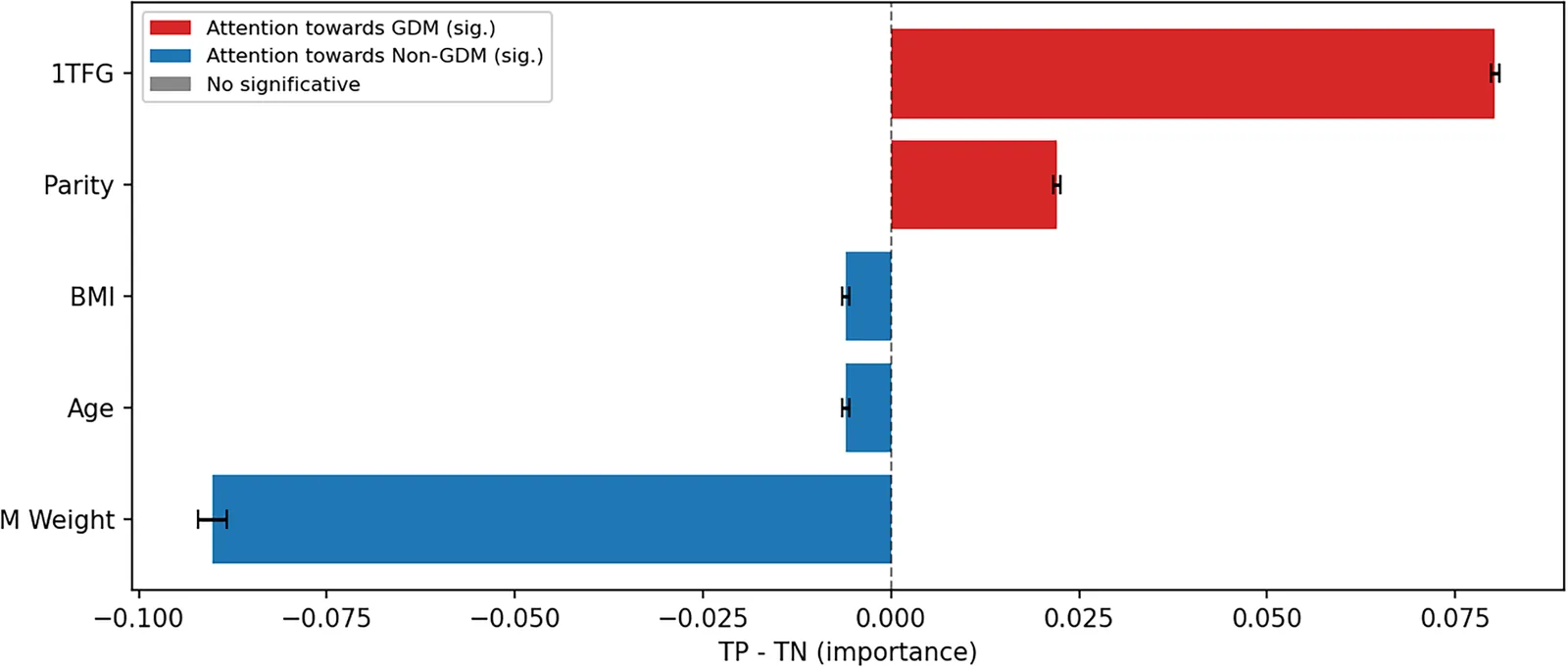
Variable-level attention contrast between TP and TN groups, computed as the bootstrap difference (TP − TN) of normalized Grad-CAM importance per variable for model 8. Error bars represent 95% confidence intervals from 1,000 bootstrap iterations resampling patients with replacement within each group. Positive values indicate variables favorably attended by the model when predicting GDM (red); negative values indicate variables preferentially attended when predicting non-GDM (blue); grey indicates non-significant contrasts whose 95% CI crosses zero. The narrow confidence intervals reflect the consistent attention patterns applied by the model across patients within each predictive group.

To complement the spatial interpretation with the underlying clinical profile of each group, **Table 6** presents the mean and standard deviation of the continuous variables in their original units for the patients in each group. Patients in the TP and FP groups exhibited similar metabolic risk profiles (mean BMI 32.40 ± 7.52 and 31.41 ± 4.99 respectively; mean 1TFG 88.50 ± 10.18 mg/dL and 85.39 ± 7.51 mg/dL respectively), both substantially elevated relative to the TN group (mean BMI 27.30 ± 5.28; mean 1TFG 74.32 ± 8.25 mg/dL). These findings suggest that FP cases were not arbitrary errors but rather patients with metabolically high-risk profiles who did not progress to GDM despite their phenotypic similarity to TP cases. Conversely, FN patients (n=7) exhibited clinical profiles closely resembling those of TN patients (mean BMI 27.47 ± 4.35; mean 1TFG 78.43 ± 6.11 mg/dL), with values that fall within the non-GDM distribution despite their eventual GDM diagnosis. This pattern suggests that these false negatives correspond to GDM cases whose risk is not captured by the routinely available clinical and demographic variables included in this study.

**Table 6:** Clinical characteristics (five continuous variables) of the TP, TN, FP, and FN populations for model 8, expressed as mean ± standard deviation.

Variable	TP (n=32)	TN (n=246)	FP (n=38)	FN (n=7)
Age	31.78 ± 6.04	26.82 ± 5.31	33.29 ± 5.91	24.29 ± 4.42
Maternal Weight	82.10 ± 19.15	69.47 ± 14.09	78.36 ± 14.27	67.51 ± 11.99
BMI	32.40 ± 7.52	27.30 ± 5.28	31.41 ± 4.99	27.47 ± 4.35
Parity	1.59 ± 1.34	0.84 ± 0.88	2.05 ± 1.77	0.57 ± 0.79
1TFG	88.50 ± 10.18	74.32 ± 8.25	85.39 ± 7.51	78.43 ± 6.11

The direction of the variable-level differences observed in the Grad-CAM contrast (TP–TN) reflects where the model preferentially allocates attention rather than the direction of the clinical effect. The direction of the clinical effect is reflected by the input distributions in: TP patients exhibited higher Maternal Weight than TN patients (82.10 ± 19.15 kg vs 69.47 ± 14.09 kg), consistent with the known association between elevated maternal weight and GDM risk. The negative TP–TN contrast for Maternal Weight in the Grad-CAM analysis indicates that the model relies more strongly on this variable when ruling out GDM than when confirming it. This pattern is consistent with the clinical observation that low maternal weight provides stronger evidence against GDM, whereas elevated maternal weight alone is insufficient for prediction given its high prevalence in the general population.

As a representation-independent control, permutation importance was computed for LR and XGBoost proxy models trained on the same five continuous variables. The two proxy models showed strong ranking agreement (Spearman ρ=0.900, p=0.037) and both ranked 1TFG first. Grad-CAM also placed 1TFG among its two largest attributions by magnitude; however, overall ranking agreement between Grad-CAM and the proxy models was low and not statistically significant (LR: ρ=-0.300, p=0.624; XGBoost: ρ=-0.100, p=0.873). The association between Grad-CAM importance and mean column position was also weak and not statistically significant (ρ=0.205, p=0.741).

## Discussion

DL models have demonstrated superior performance over traditional ML models in two-dimensional pattern recognition tasks. In this study, we proposed a method to transform tabular data (vectors) into images (matrices), enabling the application of DL models such as CNNs, which require 2D inputs, for classification and clinical prediction tasks. This approach was applied to GDM prediction and was benchmarked against previous studies using traditional ML models, including LR, MLP, and SVMs. Using the same dataset ensures a fair comparison and facilitates evaluation under real-world conditions.

The results show that the proposed model (DL Gen) achieved higher specificity than traditional ML models across the full range of sensitivities analyzed. Across the nine matched sensitivity levels, DL Gen achieved a mean absolute specificity gain of 13.93 percentage points, corresponding to an average of 39.56 additional true negatives and 39.56 fewer misclassified patients. The median relative specificity improvement was 18.88% (**Table 4**). Reducing false-positive classifications may decrease unnecessary follow-up investigations, patient anxiety, and healthcare resource utilization while maintaining the ability to identify women at increased risk of GDM. These findings indicate that the model improves early GDM prediction while relying on a limited set of routinely collected variables during standard prenatal checkups, supporting its potential for clinical implementation. The variables used in this study are routinely collected during standard medical checkups and examinations in pregnant women across diverse healthcare settings.

As shown in **Table 5**, compared with the previous DL models without GA, DL Gen achieved a mean absolute specificity gain of 9.62 percentage points across the nine matched sensitivity levels, corresponding to an average of 27.33 additional true negatives and 27.33 fewer misclassified patients. A key methodological contribution of the proposed approach is the optimization of the spatial arrangement of variables using a genetic algorithm. This flexibility allows clinicians to select operating points according to clinical priorities, balancing sensitivity and specificity according to the intended clinical application. For example, model 4 achieved a sensitivity of 92% (3 FN), a specificity of 77% (66 FP), and an AUCROC of 0.8519, which may be suitable in settings where high sensitivity is prioritized. Model 8 achieved a sensitivity of 82% (7 FN), a specificity of 87% (38 FP), and an AUCROC of 0.8331, and may be preferable in scenarios requiring a more balanced trade-off between sensitivity and specificity, such as resource-constrained healthcare settings.

The comparison against gradient boosting baselines (**Supplementary Table S3**) is consistent with our previous findings (54): XGBoost and LightGBM, two widely used gradient boosting frameworks for tabular clinical prediction, achieved performance below that of MLP and SVM on this dataset, with maximum AUC-ROC values close to 0.81 in both cases. The convergence of both frameworks to a similar performance limit suggests that this ceiling reflects an inherent limitation of tree-based ensembles applied to this low-dimensional clinical feature space, rather than an artifact of hyperparameter tuning. In contrast, DL Gen consistently exceeded this threshold across all operational sensitivity levels, supporting the hypothesis that the proposed tabular-to-image transformation enables the convolutional architecture to capture clinically relevant predictive patterns beyond what is accessible to tree-based models on the same input variables.

Beyond predictive performance, DL Gen enables early GDM detection during the first and early second trimesters of pregnancy, well ahead of the standard oral glucose tolerance test (OGTT) administered at weeks 24–28, thereby supporting timely preventive interventions, closer clinical follow-up, and potentially reducing adverse maternal and neonatal outcomes. The substantial reduction in false positives relative to prior approaches represents a clinically meaningful advance, minimizing unnecessary interventions without compromising case detection.

Out of 9 DL Gen models, 6 models used DA (1, 2, 4, 7, 8, and 9). As can be seen on **Supplementary Table S7**, models without DA (3, 5 and 6) improved an average of 13 cases. In contrast, models with DA reduced errors by an average of 63.5 patients due to the application of DA. Nevertheless, most of this enhancement comes from models 1 and 2 in which the use of DA reduced FP in more than 100 cases.

The contributions of DA and GA optimization to model performance were examined through complementary ablation analyses. As shown in **Supplementary Table S7**, within GA-optimized configurations, models using DA showed a greater reduction in classification errors than those without DA (63.5 vs. 13 patients on average), indicating a meaningful contribution of DA to model performance. To separately assess the contribution of GA optimization, we trained 50 additional models with the same architecture and DA configuration as Model 8, but with variable arrangements generated from independently sampled random genotypes. Random arrangements achieved a median test AUC-ROC of 0.723 [IQR: 0.712–0.744] and a maximum of 0.777, whereas the GA-optimized Model 8 achieved an AUC-ROC of 0.833. Thus, with DA held constant, the GA-optimized configuration achieved an AUC-ROC 0.110 higher than the median random arrangement and 0.056 higher than the best-performing random arrangement; none of the 50 random configurations reached the performance of the GA-optimized model (**Supplementary Tables S13**, **S14**). Together, these analyses indicate that DA and GA optimization make distinct contributions to model performance: DA improves performance within GA-optimized configurations, whereas GA optimization provides additional improvement beyond that achieved with DA alone.

To further assess whether the performance of the model was driven predominantly by 1TFG, we evaluated a univariate baseline using this variable alone. Logistic Regression on 1TFG achieved a test AUC-ROC of 0.785 [95% CI: 0.705–0.862]. At the same operating sensitivity as Model 8 (0.821), the univariate model achieved a specificity of 0.539 and a PPV of 0.196, compared with 0.866 and 0.457, respectively, for Model 8 (**Supplementary Tables S15**, **S16**). These results indicate that, although 1TFG contains substantial predictive information, this variable alone does not reproduce the performance of the multivariable model.

Finally, to evaluate the contribution of backbone initialization, we retrained Model 8 with randomly initialized ResNet-50 weights, with the initialization variance scaled according to the number of input and output units in each layer, instead of ImageNet-pretrained weights, while maintaining the same architecture and training configuration. The randomly initialized model achieved an AUC-ROC of 0.830 compared with 0.833 for the ImageNet-pretrained model. Sensitivity remained unchanged at 0.821, while specificity decreased from 0.866 to 0.842 and PPV decreased from 0.457 to 0.416 (**Supplementary Table S17**). These results indicate that ImageNet pretraining provides a modest performance benefit but is not the primary determinant of the observed predictive performance.

### Model interpretability and clinical coherence

Among the operating points evaluated in **Table 3**, we consider Model 8 the most appropriate configuration for routine early pregnancy GDM screening. It provides a favorable balance between sensitivity (0.821) and specificity (0.866), maintaining a high detection rate while limiting false positives and the associated follow-up burden. Although higher-sensitivity configurations identify additional GDM cases, they do so at the cost of substantially lower specificity. Model 8 also relies on five variables routinely available during early pregnancy care, supporting its potential clinical applicability. In addition, the availability of multiple operating points provides flexibility to select alternative sensitivity-specificity trade-offs according to local healthcare resources and clinical priorities, including individual patient considerations.

Beyond predictive performance, the interpretability analysis provided insight into the decision patterns learned by the DL Gen model. Grad-CAM revealed that the selected model (model 8) adopted two spatially distinct attention strategies, one associated with positive predictions and the other with negative predictions, that were largely consistent across patients within each group. This consistency suggests that the genetic algorithm converged toward image layouts in which clinically relevant variables occupy positions that the convolutional architecture can exploit in a structured and reproducible manner.

The variables differentially attended by the model when predicting GDM versus non-GDM, namely 1TFG, Parity, and Maternal Weight, are well-established risk factors for GDM in the clinical literature (5). The preferential attention to 1TFG when predicting GDM is particularly notable: the model identifies the upper end of the normoglycemic range as a strong indicator of subsequent GDM development. This observation is consistent with previous reports that glycemia values in early pregnancy, even within the normal range, predict GDM risk later in gestation (66). The preferential reliance on Maternal Weight when predicting non-GDM, rather than when predicting GDM, can be interpreted clinically: low maternal weight provides stronger evidence against GDM, whereas elevated maternal weight alone is insufficient given its high prevalence in the general pregnant population. This asymmetry reflects established clinical reasoning, in which body composition contributes more strongly to ruling out GDM than to confirming it.

Notably, Age and BMI exhibited minimal differential attention between prediction directions. In the case of BMI, this may partly reflect its dependence on maternal weight and height, although both BMI and maternal weight were independently included as input variables. This finding does not contradict the importance of these variables despite their established role as GDM risk factors and their consistent identification by feature-selection methods in our previous work (54). This finding does not contradict the importance of these variables; rather, it suggests that the model integrates them as baseline context contributing similarly to both prediction directions, while delegating discriminative capacity to variables with greater dynamic range across patients (1TFG, Parity, Maternal Weight).

The analysis of error groups yielded clinically meaningful patterns. False positive cases exhibited metabolic risk profiles comparable to true positive cases, with elevated BMI, 1TFG, and Maternal Weight relative to the non-GDM population. This indicates that false positives are not arbitrary classification errors but rather patients with metabolically high-risk profiles who did not progress to GDM despite their phenotypic similarity to confirmed cases. From a clinical screening perspective, identifying these patients as high-risk may still be clinically valuable, as they could benefit from preventive lifestyle interventions, even in the absence of GDM development (67). False negative cases, in contrast, were clinically indistinguishable from true negatives across all five continuous variables analyzed, with mean values falling within the non-GDM distribution. This pattern suggests that these GDM cases have phenotypes not captured by the routinely collected variables analyzed in this study, indicating an informational rather than methodological limitation. This finding supports the proposal in our previous work (54) that incorporating biomarkers beyond standard clinical and demographic variables may further improve early GDM prediction, particularly for patients whose risk profiles are not apparent in routine prenatal assessments.

Several limitations of the interpretability analysis should be acknowledged. Grad-CAM identifies the regions toward which the model preferentially directs attention but does not independently determine the direction of effect of individual variables on prediction; therefore, interpretation of the attention maps was complemented by the group-wise input distribution analysis presented in **Table 6**. The observed bootstrap confidence intervals reflect the consistency of attention patterns within the evaluated trained model and should not be interpreted as estimates of population-level effects or cross-model variability. In addition, the interpretability analyses were conducted within the proposed genotype-based tabular-to-image framework using the operationally selected model, and future studies should evaluate the robustness of these patterns across independent cohorts, model initializations, and alternative architectures. Nevertheless, the substantial consistency of the attention patterns across patients, together with their concordance with established clinical risk factors and clinically plausible decision logic, supports the clinical coherence of the learned representations and strengthens the biological plausibility of the proposed approach.

The representation independent permutation analyses (**Supplementary Tables S18**, **S19**) consistently identified 1TFG as the strongest global predictor, whereas the complete feature ranking showed limited agreement with Grad-CAM. This difference is relevant because permutation importance and Grad-CAM quantify different aspects of model behavior and should not be interpreted as equivalent measures of feature importance. Furthermore, no statistically significant monotonic association was observed between Grad-CAM attribution magnitude and mean variable column position. Given the small number of variables, this finding should be interpreted cautiously and does not exclude all possible effects of the image representation. Accordingly, the Grad-CAM analysis is presented as a model specific exploratory interpretation rather than definitive evidence of clinical feature importance.

The calibration analysis complements the discrimination results by showing good agreement between predicted and observed outcomes in the probability range containing most observations, while also identifying some deviation from ideal calibration. DCA further showed a higher net benefit for DL Gen 8 than for both baseline models across the evaluated clinically relevant threshold range. Together, these findings provide additional support for the potential clinical applicability of the proposed model. Nevertheless, because calibration may vary across populations and clinical settings, it should be reassessed and, if necessary, updated during prospective external validation before clinical implementation.

Because the genetic algorithm evaluated a large number of candidate configurations using a comparatively small validation set, model selection may be susceptible to validation set overfitting and selection related optimism. To assess this possibility empirically, we compared validation- and test set performance for the selected DL Gen models (**Supplementary Table S8**). The observed gaps did not show systematic validation set optimism, with test set AUC-ROC exceeding validation set AUC-ROC for all nine models. Nevertheless, the absence of an observed systematic optimistic gap does not exclude selection related bias, particularly given the limited validation set size and sampling variability between the validation and test partitions.

Since the data originated from a single institution in Chile, the performance of the model in other populations, healthcare systems, and geographic settings requires external validation. Although the study institution is one of the largest medical centers in the country and serves a heterogeneous population of more than 700,000 individuals across multiple municipalities and socioeconomic strata, this diversity does not substitute for validation in geographically and demographically distinct populations. Therefore, prospective multicenter external validation is necessary to confirm the generalizability and performance of the model before clinical implementation.

A potential limitation concerns the transportability of the model across healthcare systems and populations. Although the predictor variables are routinely collected, differences in measurement protocols, including the timing and conditions of maternal weight, BMI, and 1TFG assessment, as well as differences in the distribution of maternal characteristics and GDM prevalence across populations, may influence model calibration and predictive performance. Additionally, our cohort represents a single healthcare system; therefore, external validation in independent populations and healthcare settings with different measurement practices and population characteristics is required before broader application of the model.

A limitation of the outcome definition is that the OGTT protocol did not include the 1-hour glucose measurement required for the complete IADPSG criteria. Therefore, participants who would have met the IADPSG definition exclusively on the basis of an elevated 1-hour glucose value could have been classified as non-GDM. The number of potentially affected participants and the resulting effect on performance estimates cannot be determined from the available data. This potential outcome misclassification should therefore be considered when interpreting the results and reinforces the importance of external validation in cohorts assessed using the complete IADPSG criteria.

### Comparison with the state of the art

Comparison with other studies is challenging because they rely on different population cohorts and exhibit variations in prevalence, as well as genetic, economic, social, or geographical factors that may influence GDM prediction, even when applying the same GDM criteria [1]. Moreover, most studies do not make their models publicly available or require data types or datasets that are not accessible for comparison. Nevertheless, **Table 7** presents representative results from the state of the art that serve as a reference. The selected models are based on similar data complexity and the same or comparable GDM criteria.

**Table 7:** Results of models of the state of the art published in other studies.

Models	Accuracy	Sensitivity	Specificity	Recall macro	AUC-ROC
(15)LR	–	0.81	0.53	0.67*	0.67
(18)DNN – 7 Variables	–	0.70	0.69	0.695*	0.77
(20)LR	–	–	–	–	0.757
(21)LR	–	–	–	–	0.778
(26)LGBM – Q-based	–	–	–	–	0.799
(27)LR	0.64 -	0.58 0.66	0.80 0.75	0.69* 0.705*	0.766
(28)LR	–	–	–	–	0.70
(29)RF	–	0.651	0.813	0.732*	0.777
(35)LR – Simple	–	0.582	0.769	0.6755*	0.694
(36)LR	–	0.716	0.652	0.684*	0.728
(37)SVM – Model 1	–	0.733	0.768	0.751	0.792
(38)XGB – E0	–	–	–	–	0.720

*Values calculated by us based on results presented.

## Conclusion

Gestational diabetes mellitus remains a leading cause of adverse maternal and neonatal outcomes, with consequences extending beyond pregnancy into later life for both mother and child. Early and accurate prediction of GDM is essential to enable timely management through lifestyle modification, enhanced clinical monitoring, and, where indicated, pharmacological management.

The findings of this study demonstrate that accurate early prediction of GDM can be improved through a method that transforms tabular data into images, using a genetic algorithm to optimize the spatial arrangement of variables. This approach enables the use of DL models such as CNNs, architectures with well-established strengths in two-dimensional pattern recognition, in a domain conventionally addressed by traditional ML models with tabular data (vector inputs). By bridging tabular clinical data and deep learning, the proposed framework exploits the representational capacity of CNNs without requiring additional data collection beyond routine prenatal assessments.

Beyond predictive performance, interpretability analysis via Grad-CAM demonstrated that the attention patterns of the model align with established clinical risk factors for GDM, with first-trimester fasting glycemia and parity preferentially attended for positive predictions and maternal weight for negative predictions. The consistency of these patterns across patients supports the validity of the proposed tabular-to-image transformation framework and provides transparency into the decision logic of the model, an important requirement for clinical adoption.

The proposed model, DL Gen, achieved a mean absolute specificity gain of 13.93 percentage points across the analyzed sensitivity range (0.79–1.0) relative to traditional ML comparators and 9.62 percentage points relative to our previously published DL models without GA, corresponding to a clinically meaningful reduction in false positives and unnecessary interventions. Crucially, these gains were achieved using a limited set of variables routinely collected during standard prenatal care, supporting the generalizability and feasibility of the model for real-world implementation across diverse healthcare settings.

Taken together, these findings suggest that the proposed tabular-to-image transformation framework represents a promising direction for early GDM screening, with the potential to support clinicians in identifying at-risk individuals earlier in pregnancy than current standard-of-care approaches allow.

## Data Availability

The data analyzed in this study is subject to the following licenses/restrictions: The datasets analyzed in this study are not publicly available due to privacy restrictions associated with sensitive clinical patient data. The dataset was provided by the Hospital Parroquial de San Bernardo, Chile. Access may be granted to qualified researchers upon reasonable request and with permission from the institution. Requests to access these datasets should be directed to Sebastian Illanes, sillanes@uandes.cl.

## Glossary

1TFG: First trimester fasting glycemia
ADA: American Diabetes Association
ALT: Alanine transaminase
APTT: Activated partial thromboplastin time
AST: Aspartate aminotransferase
AUCROC: Area under the curve of receiver operating characteristic
BMI: Body mass index
BNB: Bernoulli naïve bayes
BRF: Balanced random forest
CNN: Convolutional neural network
DA: Data augmentation
DL: Deep learning
DNN: Deep neural network
DT: Decision tree
ECE: Expected calibration error
EHR: Electronic health record
ET: Extra trees
FBG: Fasting blood glycemia
FC: Fully connected
FN: False negative
FP: False positive
FPG: Fasting plasma glucose
GB: Gradient boosting
GDM: Gestational diabetes mellitus
GNB: Gaussian naïve bayes
Grad-CAM: Gradient-weighted class activation mapping
HAPO: Hyperglycemia and adverse pregnancy outcome group
HBA_1c_: Glycated hemoglobin
HDL-C: High density lipoprotein cholesterol
IADPSG: International Association of Diabetes and Pregnancy Study Groups
KNN: K-nearest neighbors
LDL-C: Low-density lipoprotein cholesterol
LGBM: Light gradient boosting machine
LR: Logistic regression
ML: Machine learning
MLP: Multi-layer perceptron
NICE: National institute for health and care excellence
OGTT: Oral glucose tolerance test
PCOS: Polycystic ovary syndrome
PPV: Positive Predictive Value
RF: Random forest
sdLDL-C: Small and dense low-density lipoprotein cholesterol
SVM: Support vector machine
TN: True negative
TG: Triglyceride
TP: True positive
TyG: Triglyceride-glucose
WHO: World Health Organization
XGB: Extreme gradient boosting
