# Supplementary material for: Early prediction of gestational diabetes mellitus using deep learning applied to routinely collected clinical data
Source: Front Endocrinol (Lausanne). 2026 Oct 5;17:1941799. doi: 10.3389/fendo.2026.1941799 (PMC13677491; doi:10.3389/fendo.2026.1941799)
Supplement: Supplementary file 1 [file SupplementaryFile1.docx]

**Supplementary Material**

**Figure S1**: Fitness evolution for the second seed. Blue indicates the best fitness, orange indicates the mean fitness of the elite group (10 individuals), and green indicates the mean fitness of the population.

**Figure S2**: Fitness evolution for the third seed. Blue indicates the best fitness, orange indicates the mean fitness of the elite group (10 individuals), and green indicates the mean fitness of the population.

**Figure S3**: Fitness evolution for the fourth seed. Blue indicates the best fitness, orange indicates the mean fitness of the elite group (10 individuals), and green indicates the mean fitness of the population.

**Figure S4**: Fitness evolution for the fifth seed. Blue indicates the best fitness, orange indicates the mean fitness of the elite group (10 individuals), and green indicates the mean fitness of the population.

**Table S1**: Hyperparameters used for each model.

| **Hyperparameter** | **Used by Models** | **Value** |
| --- | --- | --- |
| Binary Variables | D1, D2, D4, D8, D9 | Use of Alcohol, Use of Illicit drugs, Cardiac diseases, Urinary Tract Disease, Psychiatric disorders, Chronic kidney diseases, Inflammatory bowel disease, Chronic lung disease, Insulin resistance, Use of Antihypertensive drugs |
| Binary Variable | 7 | Use of Illicit drugs, Cardiac diseases, Urinary Tract Disease, Psychiatric disorders, Chronic kidney diseases, Inflammatory bowel disease, Insulin resistance, Use of Antihypertensive drugs |
| Output Layer | 1, 2, 3, 4, 5, 6, 7, D2, D3, D4, D5, D6, D7, D8, D9 | Last convolutional layer (a.k.a. ‘layer4.2.conv3’) |
| Output Layer | 8, 9, D1 | Average Pooling layer (Avgpool) |
| Optimizer | 1, 2, 3, 4, 5, 7, 8 | Adam |
| Spatial position of Variables | D1, D2, D3, D4, D5, D6, D7, D8, D9 | 3, 1, 2, 4, 0 (width 10 of each) |
| Spatial position of Variables | 1, 2, 3, 4, 5, 6, 7, 8, 9 | 0, 3, 2, 4, 3, 4, 3, 4, 1, 4, 4, 0, 4, 0, 1, 0, 1, 3, 2, 1, 3, 2, 2, 4, 2, 2, 2, 2, 4, 4, 4, 1, 4, 0, 0, 0, 4, 4, 4, 3, 4, 3, 3, 1, 3, 1, 2, 0, 1, 0 |

Variable coding:

0, age; 1, maternal weight; 2, body mass index (BMI); 3, parity; 4, first-trimester fasting glycemia.

**Table S2**: DA configuration for models of Table 3.

| **Model Number** | **Model Type** | **Variables** | **DA: %/NPP/NNP/F/R\| Range** |
| --- | --- | --- | --- |
| 1 | DL Gen | 5 | 2/1/1/No/No\| OR |
| D1 | DL | 5+10B | 0.01/10/10/No/No\| OR |
| T1 | MLP | 12 | No |
| 2 | DL Gen | 5 | 0.01/1/1/No/No\| OR |
| D2 | DL | 5+10B | 1.5/10/5/No/Yes\| OR |
| T2 | MLP | 8 | -\| LR |
| 3 | DL Gen | 5 | No/No/No/No/No\| - |
| D3 | DL | 5 | 0.1/10/10/No/Yes\| OR |
| T3 | SVM | 5 | -\| OR |
| 4 | DL Gen | 5 | 0.01/1/1/No/No\| OR |
| D4 | DL | 5+10B | 1.5/10/5/No/Yes\| OR |
| T4 | SVM | 5 | -\| OR |
| 5 | DL Gen | 5 | No/No/No/No/No\| - |
| D5 | DL | 5 | 0.1/10/5/No/No\| OR |
| T5 | MLP | 5 | -\| LR |
| 6 | DL Gen | 5 | No/No/No/No/No\| - |
| D6 | DL | 5 | 2/10/10/No/Yes\| OR |
| T6 | MLP | 7 | -\| LR |
| 7 | DL Gen | 5+8B | 0.1/1/1/No/No\| OR |
| D7 | DL | 5 | 0.1/10/5/No/Yes\| OR |
| T7 | MLP | 6 | -\| LR |
| 8 | DL Gen | 5 | 5/2/2/No/No\| OR |
| D8 | DL | 5+10B | 1/10/10/No/Yes\| OR |
| T8 | SVM | 12 | No |
| 9 | DL Gen | 5 | 5/2/2/No/No\| OR |
| D9 | DL | 5+10B | 5/10/10/No/Yes\| OR |
| T9 | SVM | 7 | -\| LR |

%: Percentual variation. NPP: New positive patients created. NNP: New negative patients created. F: Use of Vertical Flip. R: Use of random rotation. Range: Range of variation used in DA. OR: Original range. LR: Limited range. B: Binary variables. Variables: Number of Variables

**Table S3:** Results of gradient boosting baselines (XGBoost and LightGBM) tested in a previous work but not presented before [52].

| **Model Number** | **Model**  **Type** | **Variables** | **DA**  **%/NPP/NNP/F/R\| Range** | **Accuracy** | **Sensitivity** | **Specificity** | **Recall**  **Macro** | **PPV** | **AUC**  **ROC** | **TN** | **FP** | **FN** | **TP** | **FP+FN** |
| --- | --- | --- | --- | --- | --- | --- | --- | --- | --- | --- | --- | --- | --- | --- |
| - | XGB | 7 | No | 0.3560 | 1 | 0.2676 | 0.6338 | 0.1580 | 0.7890 | 76 | 208 | 0 | 39 | 208 |
| - | LGBM | 5 | No | 0.3591 | 1 | 0.2711 | 0.6356 | 0.1585 | 0.7692 | 77 | 207 | 0 | 39 | 207 |
| - | XGB | 12 | No | 0.4768 | 0.9744 | 0.4085 | 0.6914 | 0.1845 | 0.8077 | 116 | 168 | 1 | 38 | 169 |
| - | LGBM | 7 | No | 0.4799 | 0.9744 | 0.4120 | 0.6932 | 0.1854 | 0.7997 | 117 | 167 | 1 | 38 | 168 |
| - | XGB | 10 | No | 0.5356 | 0.9487 | 0.4789 | 0.7138 | 0.2000 | 0.7971 | 136 | 148 | 2 | 37 | 150 |
| - | LGBM | 5 | No | 0.5139 | 0.9487 | 0.4542 | 0.7015 | 0.1927 | 0.7874 | 129 | 155 | 2 | 37 | 157 |
| - | XGB | 10 | No | 0.5449 | 0.9231 | 0.4930 | 0.7080 | 0.2000 | 0.8087 | 140 | 144 | 3 | 36 | 147 |
| - | LGBM | 12 | No | 0.5635 | 0.9231 | 0.5141 | 0.7186 | 0.2069 | 0.7784 | 146 | 138 | 3 | 36 | 141 |
| - | XGB | 5 | No | 0.5913 | 0.8974 | 0.5493 | 0.7234 | 0.2147 | 0.7875 | 156 | 128 | 4 | 35 | 132 |
| - | LGBM | 10 | No | 0.5975 | 0.8974 | 0.5563 | 0.7269 | 0.2174 | 0.7432 | 158 | 126 | 4 | 35 | 130 |
| - | XGB | 7 | No | 0.6285 | 0.8718 | 0.5951 | 0.7334 | 0.2282 | 0.7984 | 169 | 115 | 5 | 34 | 120 |
| - | LGBM | 10 | No | 0.6594 | 0.8718 | 0.6303 | 0.7510 | 0.2446 | 0.7876 | 179 | 105 | 5 | 34 | 110 |
| - | XGB | 12 | No | 0.6563 | 0.8462 | 0.6303 | 0.7382 | 0.2391 | 0.7884 | 179 | 105 | 6 | 33 | 111 |
| - | LGBM | 10 | No | 0.6378 | 0.8462 | 0.6092 | 0.7277 | 0.2292 | 0.7651 | 173 | 111 | 6 | 33 | 117 |
| - | XGB | 7 | No | 0.6625 | 0.8205 | 0.6408 | 0.7307 | 0.2388 | 0.7809 | 182 | 102 | 7 | 32 | 109 |
| - | LGBM | 10 | No | 0.6904 | 0.8205 | 0.6725 | 0.7465 | 0.2560 | 0.7539 | 191 | 93 | 7 | 32 | 100 |
| - | XGB | 5 | No | 0.6533 | 0.7949 | 0.6338 | 0.7143 | 0.2296 | 0.7770 | 180 | 104 | 8 | 31 | 112 |
| - | LGBM | 10 | No | 0.6811 | 0.7949 | 0.6655 | 0.7302 | 0.2460 | 0.7700 | 189 | 95 | 8 | 31 | 103 |

%: Percentual variation. NPP: New positive patients created. NNP: New negative patients created. F: Use of Vertical Flip. R: Use of random rotation. Range: Range of variation used in DA. OR: Original range. LR: Limited range. B: Binary variables. Variables: Number of Variables

**Table S4**: Performance of traditional machine learning models without optimization procedures (variable selection, data augmentation, or hyperparameter tuning).

| **Model Number** | **Model**  **Type** | **Vars** | **Image**  **Size** | **DA**  **%/NPP/NNP/F/R\| Range** | **Accuracy** | **Sensitivity** | **Specificity** | **Recall**  **Macro** | **AUC**  **ROC** | **TN** | **FP** | **FN** | **TP** | **FP+FN** |
| --- | --- | --- | --- | --- | --- | --- | --- | --- | --- | --- | --- | --- | --- | --- |
| NO1 | MLP | 31 | - | No | 0.2941 | 1 | 0.1972 | 0.5986 | 0.7124 | 56 | 228 | 0 | 39 | 228 |
| NO2 | MLP | 31 | - | No | 0.3406 | 0.9744 | 0.2535 | 0.6139 | 0.7124 | 72 | 212 | 1 | 38 | 213 |
| NO3 | MLP | 31 | - | No | 0.3808 | 0.9487 | 0.3028 | 0.6258 | 0.7124 | 86 | 198 | 2 | 37 | 200 |
| NO4 | MLP | 31 | - | No | 0.3963 | 0.9231 | 0.3239 | 0.6235 | 0.7124 | 92 | 192 | 3 | 36 | 195 |
| NO5 | MLP | 31 | - | No | 0.4365 | 0.8974 | 0.3732 | 0.6353 | 0.7124 | 106 | 178 | 4 | 35 | 182 |
| NO6 | MLP | 31 | - | No | 0.5046 | 0.8718 | 0.4542 | 0.6630 | 0.7124 | 129 | 155 | 5 | 34 | 160 |
| NO7 | MLP | 31 | - | No | 0.5046 | 0.8462 | 0.4577 | 0.6520 | 0.7124 | 130 | 154 | 6 | 33 | 160 |
| NO8 | MLP | 31 | - | No | 0.5139 | 0.8205 | 0.4718 | 0.6462 | 0.7124 | 134 | 150 | 7 | 32 | 157 |
| NO9 | MLP | 31 | - | No | 0.5325 | 0.7949 | 0.4965 | 0.6457 | 0.7124 | 141 | 143 | 8 | 31 | 151 |

Abbreviations: %, percentage variation; NPP, new positive patients created; NNP, new negative patients created; F, vertical flip applied; R, random rotation applied; Range, variation range used in data augmentation (DA); OR, original range; LR, limited range; B, binary variables; Vars, number of variables.

**Table S5**: Performance of the proposed model (DL Gen) with different image-size hyperparameter settings in the validation set.

| **Model Number** | **Model Type** | **Number of Variables** | **Image Size** | **DA**  **%/NPP/NNP/F/R\| Range** | **AUCROC Val Set** | **Best Recall Macro Val Set** |
| --- | --- | --- | --- | --- | --- | --- |
| **1** | **DL Gen** | **5** | **250x50** | **2/1/1/No/No\| OR** | **0.8272** | **0.8245** |
| - | DL Gen | 5 | 300x50 | 2/2/1/No/No\| OR | 0.7563 | 0.7630 |
| - | DL Gen | 5+8B | 50x50 | 5/2/1/No/No\| OR | 0.7399 | 0.7341 |
| - | DL Gen | 5+8B | 1x50 | 5/1/0/No/No\| OR | 0.7745 | 0.7377 |
| - | DL Gen | 5+8B | 250x180 | 2/5/2/No/No\| OR | 0.7957 | 0.7920 |
| - | DL Gen | 5 | 250x25 | 0.1/5/5/No/No\| OR | 0.7578 | 0.7522 |
| - | DL Gen | 5+10B | 250x5 | 0.1/10/5/No/No\| OR | 0.7408 | 0.7486 |
| **2** | **DL Gen** | **5** | **250x50** | **0.01/1/1/No/No\| OR** | **0.8136** | **0.8209** |
| - | DL Gen | 5+10B | 300x50 | 1.5/2/1/No/Yes\| OR | 0.7783 | 0.7630 |
| - | DL Gen | 5+8B | 50x50 | 5/2/1/No/No\| OR | 0.7506 | 0.7558 |
| - | DL Gen | 5 | 1x50 | 5/2/1/No/No\| OR | 0.7953 | 0.7558 |
| - | DL Gen | 5+8B | 250x180 | 5/1/0/No/No\| OR | 0.7856 | 0.7739 |
| - | DL Gen | 5+10B | 250x25 | 5/5/2/No/No\| OR | 0.7462 | 0.7341 |
| - | DL Gen | 5 | 250x5 | 1/1/0/No/Yes\| OR | 0.7065 | 0.7377 |
| 3 | DL Gen | 5 | 250x50 | No/No/No/No/No\| - | **0.8149** | 0.8064 |
| - | DL Gen | 5+8B | 300x50 | 5/1/0/No/No\| OR | 0.7865 | 0.7703 |
| - | DL Gen | 5 | 50x50 | 1.5/10/10/No/Yes\| OR | 0.7059 | 0.7558 |
| - | DL Gen | 5 | 1x50 | 5/2/2/No/Yes\| OR | 0.7711 | 0.7486 |
| - | DL Gen | 5 | 250x180 | 1.5/5/2/No/No\| OR | 0.7733 | 0.7848 |
| - | DL Gen | 5 | 250x25 | 1.5/10/10/No/Yes\| OR | 0.7572 | 0.7341 |
| - | DL Gen | 5 | 250x5 | 1/5/2/No/No\| OR | 0.7890 | **0.8065** |
| 4 | DL Gen | 5 | 250x50 | 0.01/1/1/No/No\| OR | **0.8048** | 0.8028 |
| - | DL Gen | 5 | 300x50 | 0.01/1/0/No/No\| OR | 0.7752 | 0.7486 |
| - | DL Gen | 5 | 50x50 | 0.1/1/1/No/No\| OR | 0.7144 | 0.7449 |
| - | DL Gen | 5 | 1x50 | 0.1/5/2/No/No\| OR | 0.7632 | 0.7377 |
| - | DL Gen | 5+8B | 250x180 | 1/2/2/No/Yes\| OR | 0.7607 | 0.7703 |
| - | DL Gen | 5 | 250x25 | 5/5/2/No/No\| OR | 0.7226 | 0.7159 |
| - | DL Gen | 5 | 250x5 | 1/5/2/No/No\| OR | 0.7890 | **0.8065** |

Table S5 (Continued)

| **Model Number** | **Model Type** | **Number of Variables** | **Image Size** | **DA**  **%/NPP/NNP/F/R\| Range** | **AUCROC Val Set** | **Best Recall Macro Val Set** |
| --- | --- | --- | --- | --- | --- | --- |
| 5 | DL Gen | 5 | 250x50 | No/No/No/No/No\| - | **0.7931** | **0.7956** |
| - | DL Gen | 5 | 300x50 | 5/1/0/No/No\| OR | 0.7834 | 0.7449 |
| - | DL Gen | 5 | 50x50 | 0.01/1/0/No/No\| OR | 0.7074 | 0.7594 |
| - | DL Gen | 5 | 1x50 | 0.1/5/2/No/No\| OR | 0.7632 | 0.7377 |
| - | DL Gen | 5+8B | 250x180 | 0.01/2/2/No/Yes\| OR | 0.7793 | 0.7775 |
| - | DL Gen | 5 | 250x25 | 5/5/2/No/No\| OR | 0.7226 | 0.7159 |
| - | DL Gen | 5 | 250x5 | 0.1/5/2/No/Yes\| OR | 0.7487 | 0.7449 |
| 6 | DL Gen | 5 | 250x50 | No/No/No/No/No\| - | 0.7912 | 0.8064 |
| - | DL Gen | 5 | 300x50 | 1.5/1/1/No/No\| OR | 0.778 | 0.7486 |
| - | DL Gen | 5+10B | 50x50 | No/No/No/No/Yes\| - | 0.7106 | 0.7413 |
| - | DL Gen | 5 | 1x50 | 1/1/0/No/No\| OR | 0.7563 | 0.7196 |
| - | DL Gen | 5 | 250x180 | 5/2/1/No/Yes\| OR | 0.6923 | 0.7232 |
| - | DL Gen | 5+8B | 250x25 | 5/2/2/No/No\| OR | **0.8051** | **0.8101** |
| - | DL Gen | 5 | 250x5 | 1.5/1/0/No/No\| OR | 0.7267 | 0.763 |
| 7 | DL Gen | 5+8B | 250x50 | 0.1/1/1/No/No\| OR | **0.7991** | **0.8028** |
| - | DL Gen | 5 | 300x50 | 0.1/1/1/No/No\| OR | 0.7424 | 0.7413 |
| - | DL Gen | 5+10B | 50x50 | No/No/No/No/Yes\| - | 0.7106 | 0.7413 |
| - | DL Gen | 5 | 1x50 | 1/1/0/No/No\| OR | 0.7563 | 0.7196 |
| - | DL Gen | 5+10B | 250x180 | 0.1/2/1/No/Yes\| OR | 0.7374 | 0.7449 |
| - | DL Gen | 5+8B | 250x25 | 5/2/2/No/No\| OR | 0.7815 | 0.7594 |
| - | DL Gen | 5 | 250x5 | 1.5/1/0/No/No\| OR | 0.7267 | 0.763 |
| 8 | DL Gen | 5 | 250x50 | 5/2/2/No/No\| OR | 0.7292 | **0.7883** |
| - | DL Gen | 5 | 300x50 | 1.5/10/10/No/Yes\| OR | 0.732 | 0.7377 |
| - | DL Gen | 5 | 50x50 | 2/2/2/No/Yes\| OR | 0.721 | 0.7703 |
| - | DL Gen | 5 | 1x50 | 1/1/0/No/No\| OR | 0.7563 | 0.7196 |
| - | DL Gen | 5 | 250x180 | 1/2/2/No/Yes\| OR | 0.7767 | 0.7848 |
| - | DL Gen | 5+8B | 250x25 | 0.1/1/0/No/Yes\| OR | **0.8152** | 0.7667 |
| - | DL Gen | 5 | 250x5 | 2/5/5/No/Yes\| OR | 0.7474 | 0.7812 |

Table S5 (Continued)

| **Model Number** | **Model Type** | **Number of Variables** | **Image Size** | **DA**  **%/NPP/NNP/F/R\| Range** | **AUCROC Val Set** | **Best Recall Macro Val Set** |
| --- | --- | --- | --- | --- | --- | --- |
| 9 | DL Gen | 5 | 250x50 | 5/2/2/No/No\| OR | 0.7314 | 0.7811 |
| - | DL Gen | 5 | 300x50 | 1/10/10/No/Yes\| OR | 0.727 | 0.7486 |
| - | DL Gen | 5 | 50x50 | 1.5/1/0/No/No\| OR | 0.7333 | 0.7558 |
| - | DL Gen | 5+8B | 1x50 | 0.1/2/2/No/No\| OR | 0.7251 | 0.7159 |
| - | DL Gen | 5 | 250x180 | 0.01/2/No/Yes\| OR | 0.7635 | 0.7775 |
| - | DL Gen | 5 | 250x25 | 5/10/10/No/Yes\| OR | 0.7739 | 0.7413 |
| - | DL Gen | 5 | 250x5 | 2/1/0/No/Yes\| OR | **0.8023** | **0.8029** |

**Table S6**: Performance of the proposed model (DL Gen) with different image-size hyperparameter settings in the test set.

| **Model Number** | **Model**  **Type** | **Vars** | **Image**  **Size** | **DA**  **%/NPP/NNP/F/R\| Range** | **Accuracy** | **Sensitivity** | **Specificity** | **Recall**  **Macro** | **AUC**  **ROC** | **TN** | **FP** | **FN** | **TP** | **FP+FN** |
| --- | --- | --- | --- | --- | --- | --- | --- | --- | --- | --- | --- | --- | --- | --- |
| **1** | **DL Gen** | **5** | **250x50** | **2/1/1/No/No\| OR** | **0.6037** | **1** | **0.5493** | **0.7746** | **0.8414** | **156** | **128** | **0** | **39** | **128** |
| - | DL Gen | 5 | 300x50 | 2/2/1/No/No\| OR | 0.5542 | 1 | 0.4930 | 0.7465 | 0.8251 | 140 | 144 | 0 | 39 | 144 |
| - | DL Gen | 5+8B | 50x50 | 5/2/1/No/No\| OR | 0.5697 | 1 | 0.5106 | 0.7553 | 0.8080 | 145 | 139 | 0 | 39 | 139 |
| - | DL Gen | 5+8B | 1x50 | 5/1/0/No/No\| OR | 0.4334 | 1 | 0.3556 | 0.6778 | 0.8059 | 101 | 183 | 0 | 39 | 183 |
| - | DL Gen | 5+8B | 250x180 | 2/5/2/No/No\| OR | 0.4427 | 1 | 0.3662 | 0.6831 | 0.7833 | 104 | 180 | 0 | 39 | 180 |
| - | DL Gen | 5 | 250x25 | 0.1/5/5/No/No\| OR | 0.3963 | 1 | 0.3134 | 0.6567 | 0.7031 | 89 | 195 | 0 | 39 | 195 |
| - | DL Gen | 5+10B | 250x5 | 0.1/10/5/No/No\| OR | 0.4427 | 1 | 0.3662 | 0.6831 | 0.7453 | 104 | 180 | 0 | 39 | 180 |
| 2 | DL Gen | 5 | 250x50 | 0.01/1/1/No/No\| OR | 0.6316 | 0.9744 | 0.5845 | 0.7794 | 0.8329 | 166 | 118 | 1 | 38 | 119 |
| - | DL Gen | 5+10B | 300x50 | 1.5/2/1/No/Yes\| OR | 0.6502 | 0.9744 | 0.6056 | 0.7900 | 0.8367 | 172 | 112 | 1 | 38 | 113 |
| - | DL Gen | 5+8B | 50x50 | 5/2/1/No/No\| OR | 0.5759 | 0.9744 | 0.5211 | 0.7477 | 0.8120 | 148 | 136 | 1 | 38 | 137 |
| - | DL Gen | 5 | 1x50 | 5/2/1/No/No\| OR | 0.5356 | 0.9744 | 0.4754 | 0.7249 | 0.8105 | 135 | 149 | 1 | 38 | 150 |
| **-** | **DL Gen** | **5+8B** | **250x180** | **5/1/0/No/No\| OR** | **0.6625** | **0.9744** | **0.6197** | **0.7970** | **0.8081** | **176** | **108** | **1** | **38** | **109** |
| - | DL Gen | 5+10B | 250x25 | 5/5/2/No/No\| OR | 0.4923 | 0.9744 | 0.4261 | 0.7002 | 0.7122 | 121 | 163 | 1 | 38 | 164 |
| - | DL Gen | 5 | 250x5 | 1/1/0/No/Yes\| OR | 0.5201 | 0.9744 | 0.4578 | 0.7161 | 0.7230 | 130 | 150 | 1 | 38 | 151 |

Abbreviations: %, percentage variation; NPP, new positive patients created; NNP, new negative patients created; F, vertical flip applied; R, random rotation applied; Range, variation range used in data augmentation (DA); OR, original range; LR, limited range; B, binary variables; Vars, number of variables.

Table S6 (Continued)

| **Model Number** | **Model**  **Type** | **Vars** | **Image**  **Size** | **DA**  **%/NPP/NNP/F/R\| Range** | **Accuracy** | **Sensitivity** | **Specificity** | **Recall**  **Macro** | **AUC**  **ROC** | **TN** | **FP** | **FN** | **TP** | **FP+FN** |
| --- | --- | --- | --- | --- | --- | --- | --- | --- | --- | --- | --- | --- | --- | --- |
| 3 | DL Gen | 5 | 250x50 | No/No/No/No/No\| - | 0.6718 | 0.9487 | 0.6338 | 0.7913 | 0.8318 | 180 | 104 | 2 | 37 | 106 |
| **-** | **DL Gen** | **5+8B** | **300x50** | **5/1/0/No/No\| OR** | **0.7121** | **0.9487** | **0.6796** | **0.8142** | **0.8298** | **193** | **91** | **2** | **37** | **93** |
| - | DL Gen | 5 | 50x50 | 1.5/10/10/No/Yes\| OR | 0.6409 | 0.9487 | 0.5986 | 0.7737 | 0.8348 | 170 | 114 | 2 | 37 | 116 |
| - | DL Gen | 5 | 1x50 | 5/2/2/No/Yes\| OR | 0.5913 | 0.9487 | 0.5423 | 0.7455 | 0.8276 | 154 | 130 | 2 | 37 | 132 |
| - | DL Gen | 5 | 250x180 | 1.5/5/2/No/No\| OR | 0.6687 | 0.9487 | 0.6303 | 0.7895 | 0.8075 | 179 | 105 | 2 | 37 | 107 |
| - | DL Gen | 5 | 250x25 | 1.5/10/10/No/Yes\| OR | 0.5635 | 0.9487 | 0.5106 | 0.7296 | 0.7439 | 145 | 139 | 2 | 37 | 141 |
| - | DL Gen | 5 | 250x5 | 1/5/2/No/No\| OR | 0.6006 | 0.9487 | 0.5528 | 0.7508 | 0.7476 | 157 | 127 | 2 | 37 | 129 |
| **4** | **DL Gen** | **5** | **250x50** | **0.01/1/1/No/No\| OR** | **0.7864** | **0.9231** | **0.7676** | **0.8453** | **0.8519** | **218** | **66** | **3** | **36** | **69** |
| - | DL Gen | 5 | 300x50 | 0.01/1/0/No/No\| OR | 0.7647 | 0.9231 | 0.7430 | 0.8330 | 0.8340 | 211 | 73 | 3 | 36 | 76 |
| - | DL Gen | 5 | 50x50 | 0.1/1/1/No/No\| OR | 0.6718 | 0.9231 | 0.6373 | 0.7802 | 0.8255 | 181 | 103 | 3 | 36 | 106 |
| - | DL Gen | 5 | 1x50 | 0.1/5/2/No/No\| OR | 0.6471 | 0.9231 | 0.6092 | 0.7661 | 0.8112 | 173 | 111 | 3 | 36 | 114 |
| - | DL Gen | 5+8B | 250x180 | 1/2/2/No/Yes\| OR | 0.7121 | 0.9231 | 0.6831 | 0.8031 | 0.8151 | 194 | 90 | 3 | 36 | 93 |
| - | DL Gen | 5 | 250x25 | 5/5/2/No/No\| OR | 0.5604 | 0.9231 | 0.5106 | 0.7168 | 0.7282 | 145 | 139 | 3 | 36 | 142 |
| - | DL Gen | 5 | 250x5 | 1/5/2/No/No\| OR | 0.6130 | 0.9231 | 0.5704 | 0.7468 | 0.7476 | 162 | 122 | 3 | 36 | 125 |

Abbreviations: %, percentage variation; NPP, new positive patients created; NNP, new negative patients created; F, vertical flip applied; R, random rotation applied; Range, variation range used in data augmentation (DA); OR, original range; LR, limited range; B, binary variables; Vars, number of variables.

Table S6 (Continued)

| **Model Number** | **Model**  **Type** | **Vars** | **Image**  **Size** | **DA**  **%/NPP/NNP/F/R\| Range** | **Accuracy** | **Sensitivity** | **Specificity** | **Recall**  **Macro** | **AUC**  **ROC** | **TN** | **FP** | **FN** | **TP** | **FP+FN** |
| --- | --- | --- | --- | --- | --- | --- | --- | --- | --- | --- | --- | --- | --- | --- |
| **5** | **DL Gen** | **5** | **250x50** | **No/No/No/No/No\| -** | **0.7895** | **0.8974** | **0.7746** | **0.8360** | **0.8422** | **220** | **64** | **4** | **35** | **68** |
| - | DL Gen | 5 | 300x50 | 5/1/0/No/No\| OR | 0.7864 | 0.8974 | 0.7711 | 0.8343 | 0.8304 | 219 | 65 | 4 | 35 | 69 |
| - | DL Gen | 5 | 50x50 | 0.01/1/0/No/No\| OR | 0.7059 | 0.8974 | 0.6796 | 0.8248 | 0.8248 | 193 | 91 | 4 | 35 | 95 |
| - | DL Gen | 5 | 1x50 | 0.1/5/2/No/No\| OR | 0.6873 | 0.8974 | 0.6585 | 0.7779 | 0.8112 | 187 | 97 | 4 | 35 | 101 |
| - | DL Gen | 5+8B | 250x180 | 0.01/2/2/No/Yes\| OR | 0.7152 | 0.8974 | 0.6901 | 0.7938 | 0.8100 | 196 | 88 | 4 | 35 | 92 |
| - | DL Gen | 5 | 250x25 | 5/5/2/No/No\| OR | 0.5759 | 0.8974 | 0.5317 | 0.7146 | 0.7282 | 151 | 133 | 4 | 35 | 137 |
| - | DL Gen | 5 | 250x5 | 0.1/5/2/No/Yes\| OR | 0.6347 | 0.8974 | 0.5986 | 0.7480 | 0.7524 | 170 | 114 | 4 | 35 | 118 |
| **6** | **DL Gen** | **5** | **250x50** | **No/No/No/No/No\| -** | **0.8050** | **0.8718** | **0.7958** | **0.8338** | **0.8470** | **226** | **58** | **5** | **34** | **63** |
| - | DL Gen | 5 | 300x50 | 1.5/1/1/No/No\| OR | 0.7926 | 0.8718 | 0.7817 | 0.8267 | 0.8268 | 222 | 62 | 5 | 34 | 67 |
| - | DL Gen | 5+10B | 50x50 | No/No/No/No/Yes\| - | 0.7307 | 0.8718 | 0.7113 | 0.7915 | 0.8063 | 202 | 82 | 5 | 34 | 87 |
| - | DL Gen | 5 | 1x50 | 1/1/0/No/No\| OR | 0.7028 | 0.8718 | 0.6796 | 0.7757 | 0.8052 | 193 | 91 | 5 | 34 | 96 |
| - | DL Gen | 5 | 250x180 | 5/2/1/No/Yes\| OR | 0.7461 | 0.8718 | 0.7289 | 0.8003 | 0.7776 | 207 | 77 | 5 | 34 | 82 |
| - | DL Gen | 5+8B | 250x25 | 5/2/2/No/No\| OR | 0.5697 | 0.8718 | 0.5282 | 0.7000 | 0.6986 | 150 | 134 | 5 | 34 | 139 |
| - | DL Gen | 5 | 250x5 | 1.5/1/0/No/No\| OR | 0.6625 | 0.8718 | 0.6338 | 0.7528 | 0.7311 | 180 | 104 | 5 | 34 | 109 |

Abbreviations: %, percentage variation; NPP, new positive patients created; NNP, new negative patients created; F, vertical flip applied; R, random rotation applied; Range, variation range used in data augmentation (DA); OR, original range; LR, limited range; B, binary variables; Vars, number of variables.

Table S6 (Continued)

| **Model Number** | **Model**  **Type** | **Vars** | **Image**  **Size** | **DA**  **%/NPP/NNP/F/R\| Range** | **Accuracy** | **Sensitivity** | **Specificity** | **Recall**  **Macro** | **AUC**  **ROC** | **TN** | **FP** | **FN** | **TP** | **FP+FN** |
| --- | --- | --- | --- | --- | --- | --- | --- | --- | --- | --- | --- | --- | --- | --- |
| **7** | **DL Gen** | **5+8B** | **250x50** | **0.1/1/1/No/No\| OR** | **0.8235** | **0.8462** | **0.8204** | **0.8333** | **0.8425** | **233** | **51** | **6** | **33** | **57** |
| - | DL Gen | 5 | 300x50 | 0.1/1/1/No/No\| OR | 0.8112 | 0.8462 | 0.8063 | 0.8263 | 0.8359 | 229 | 55 | 6 | 33 | 61 |
| - | DL Gen | 5+10B | 50x50 | No/No/No/No/Yes\| - | 0.7338 | 0.8462 | 0.7183 | 0.7822 | 0.8063 | 204 | 80 | 6 | 33 | 86 |
| - | DL Gen | 5 | 1x50 | 1/1/0/No/No\| OR | 0.7461 | 0.8462 | 0.7324 | 0.7893 | 0.8052 | 208 | 76 | 6 | 33 | 82 |
| - | DL Gen | 5+10B | 250x180 | 0.1/2/1/No/Yes\| OR | 0.7585 | 0.8462 | 0.7465 | 0.7963 | 0.7772 | 212 | 72 | 6 | 33 | 78 |
| - | DL Gen | 5+8B | 250x25 | 5/2/2/No/No\| OR | 0.6192 | 0.8462 | 0.5880 | 0.7171 | 0.7227 | 167 | 117 | 6 | 33 | 123 |
| - | DL Gen | 5 | 250x5 | 1.5/1/0/No/No\| OR | 0.6935 | 0.8462 | 0.6725 | 0.7593 | 0.7311 | 191 | 93 | 6 | 33 | 99 |
| **8** | **DL Gen** | **5** | **250x50** | **5/2/2/No/No\| OR** | **0.8607** | **0.8205** | **0.8662** | **0.8434** | **0.8331** | **246** | **38** | **7** | **32** | **45** |
| **-** | **DL Gen** | **5** | **300x50** | **1.5/10/10/No/Yes\| OR** | **0.8607** | **0.8205** | **0.8662** | **0.8434** | **0.8244** | **246** | **38** | **7** | **32** | **45** |
| - | DL Gen | 5 | 50x50 | 2/2/2/No/Yes\| OR | 0.7616 | 0.8205 | 0.7535 | 0.7870 | 0.8136 | 214 | 70 | 7 | 32 | 77 |
| - | DL Gen | 5 | 1x50 | 1/1/0/No/No\| OR | 0.7554 | 0.8205 | 0.7465 | 0.7835 | 0.8052 | 212 | 72 | 7 | 32 | 79 |
| - | DL Gen | 5 | 250x180 | 1/2/2/No/Yes\| OR | 0.7647 | 0.8205 | 0.7570 | 0.7888 | 0.8073 | 215 | 69 | 7 | 32 | 76 |
| - | DL Gen | 5+8B | 250x25 | 0.1/1/0/No/Yes\| OR | 0.6316 | 0.8205 | 0.6056 | 0.7131 | 0.7175 | 172 | 112 | 7 | 32 | 119 |
| - | DL Gen | 5 | 250x5 | 2/5/5/No/Yes\| OR | 0.6935 | 0.8205 | 0.6761 | 0.7483 | 0.7432 | 192 | 92 | 7 | 32 | 99 |

Abbreviations: %, percentage variation; NPP, new positive patients created; NNP, new negative patients created; F, vertical flip applied; R, random rotation applied; Range, variation range used in data augmentation (DA); OR, original range; LR, limited range; B, binary variables; Vars, number of variables.

Table S6 (Continued)

| **Model Number** | **Model**  **Type** | **Vars** | **Image**  **Size** | **DA**  **%/NPP/NNP/F/R\| Range** | **Accuracy** | **Sensitivity** | **Specificity** | **Recall**  **Macro** | **AUC**  **ROC** | **TN** | **FP** | **FN** | **TP** | **FP+FN** |
| --- | --- | --- | --- | --- | --- | --- | --- | --- | --- | --- | --- | --- | --- | --- |
| **9** | **DL Gen** | **5** | **250x50** | **5/2/2/No/No\| OR** | **0.8514** | **0.7949** | **0.8592** | **0.8270** | **0.8164** | **244** | **40** | **8** | **31** | **48** |
| - | DL Gen | 5 | 300x50 | 1/10/10/No/Yes\| OR | 0.8483 | 0.7949 | 0.8556 | 0.8253 | 0.8107 | 243 | 41 | 8 | 31 | 49 |
| - | DL Gen | 5 | 50x50 | 1.5/1/0/No/No\| OR | 0.7740 | 0.7949 | 0.7711 | 0.7830 | 0.8233 | 219 | 65 | 8 | 31 | 73 |
| - | DL Gen | 5+8B | 1x50 | 0.1/2/2/No/No\| OR | 0.7771 | 0.7949 | 0.7747 | 0.7848 | 0.8059 | 220 | 64 | 8 | 31 | 72 |
| - | DL Gen | 5 | 250x180 | 0.01/2/No/Yes\| OR | 0.7678 | 0.7949 | 0.7641 | 0.7795 | 0.8056 | 217 | 67 | 8 | 31 | 75 |
| - | DL Gen | 5 | 250x25 | 5/10/10/No/Yes\| OR | 0.6564 | 0.7949 | 0.6373 | 0.7161 | 0.7435 | 181 | 103 | 8 | 31 | 111 |
| - | DL Gen | 5 | 250x5 | 2/1/0/No/Yes\| OR | 0.7152 | 0.7949 | 0.7042 | 0.7496 | 0.7566 | 200 | 84 | 8 | 31 | 92 |

Abbreviations: %, percentage variation; NPP, new positive patients created; NNP, new negative patients created; F, vertical flip applied; R, random rotation applied; Range, variation range used in data augmentation (DA); OR, original range; LR, limited range; B, binary variables; Vars, number of variables.

**Table S7**: Results of the proposed model (DL Gen) with and without data augmentation (DA).

| **Model Number** | **Model**  **Type** | **Variables** | **Image**  **Size** | **DA**  **%/NPP/NNP/F/R\| Range** | **Accuracy** | **Sensitivity** | **Specificity** | **Recall**  **Macro** | **AUC**  **ROC** | **TN** | **FP** | **FN** | **TP** | **FP+FN** |
| --- | --- | --- | --- | --- | --- | --- | --- | --- | --- | --- | --- | --- | --- | --- |
| **1** | **DL Gen** | **5** | **250x50** | **2/1/1/No/No\| OR** | **0.6037** | **1** | **0.5493** | **0.7746** | **0.8414** | **156** | **128** | **0** | **39** | **128** |
| 1 No DA | DL Gen | 5 | 250x50 | No/No/No/No/No\| - | 0.2291 | 1 | 0.1232 | 0.5616 | 0.8013 | 35 | 249 | 0 | 39 | 249 |
| **2** | **DL Gen** | **5** | **250x50** | **0.01/1/1/No/No\| OR** | **0.6316** | **0.9744** | **0.5845** | **0.7794** | **0.8329** | **166** | **118** | **1** | **38** | **119** |
| 2 No DA | DL Gen | 5 | 250x50 | No/No/No/No/No\| - | 0.3220 | 0.9744 | 0.2324 | 0.6034 | 0.8422 | 66 | 218 | 1 | 38 | 219 |
| **3** | **DL Gen** | **5** | **250x50** | **No/No/No/No/No\| -** | **0.6718** | **0.9487** | **0.6338** | **0.7913** | **0.8318** | **180** | **104** | **2** | **37** | **106** |
| 3 DA | DL Gen | 5 | 250x50 | 2/2/1/No/No\| OR | 0.5851 | 0.9487 | 0.5352 | 0.7420 | 0.8304 | 152 | 132 | 2 | 37 | 134 |
| **4** | **DL Gen** | **5** | **250x50** | **0.01/1/1/No/No\| OR** | **0.7864** | **0.9231** | **0.7676** | **0.8453** | **0.8519** | **218** | **66** | **3** | **36** | **69** |
| 4 No DA | DL Gen | 5 | 250x50 | No/No/No/No/No\| - | 0.6935 | 0.9231 | 0.6620 | 0.7925 | 0.8588 | 188 | 96 | 3 | 36 | 99 |
| **5** | **DL Gen** | **5** | **250x50** | **No/No/No/No/No\| -** | **0.7895** | **0.8974** | **0.7746** | **0.8360** | **0.8422** | **220** | **64** | **4** | **35** | **68** |
| 5 DA | DL Gen | 5 | 250x50 | 0.1/2/2/No/No\| OR | 0.7647 | 0.8974 | 0.7465 | 0.8220 | 0.8462 | 212 | 72 | 4 | 35 | 76 |
| **6** | **DL Gen** | **5** | **250x50** | **No/No/No/No/No\| -** | **0.8050** | **0.8718** | **0.7958** | **0.8338** | **0.8470** | **226** | **58** | **5** | **34** | **63** |
| 6 DA | DL Gen | 5 | 250x50 | 0.01/2/2/No/No\| - | 0.7926 | 0.8718 | 0.7817 | 0.8267 | 0.8453 | 222 | 62 | 5 | 34 | 67 |
| **7** | **DL Gen** | **5+8B** | **250x50** | **0.1/1/1/No/No\| OR** | **0.8235** | **0.8462** | **0.8204** | **0.8333** | **0.8425** | **233** | **51** | **6** | **33** | **57** |
| 7 No DA | DL Gen | 5+8B | 250x50 | No/No/No/No/No\| - | 0.7616 | 0.8462 | 0.7500 | 0.7981 | 0.8457 | 213 | 71 | 6 | 33 | 77 |

Abbreviations: %, percentage variation; NPP, new positive patients created; NNP, new negative patients created; F, vertical flip applied; R, random rotation applied; Range, variation range used in data augmentation (DA); OR, original range; LR, limited range; B, binary variables; Vars, number of variables.

**Table S7** (Continued)

| **Model Number** | **Model**  **Type** | **Variables** | **Image**  **Size** | **DA**  **%/NPP/NNP/F/R\| Range** | **Accuracy** | **Sensitivity** | **Specificity** | **Recall**  **Macro** | **AUC**  **ROC** | **TN** | **FP** | **FN** | **TP** | **FP+FN** |
| --- | --- | --- | --- | --- | --- | --- | --- | --- | --- | --- | --- | --- | --- | --- |
| **8** | **DL Gen** | **5** | **250x50** | **5/2/2/No/No\| OR** | **0.8607** | **0.8205** | **0.8662** | **0.8434** | **0.8331** | **246** | **38** | **7** | **32** | **45** |
| 8 No DA | DL Gen | 5 | 250x50 | No/No/No/No/No\| - | 0.7337 | 0.8205 | 0.7218 | 0.7712 | 0.7951 | 205 | 79 | 7 | 32 | 86 |
| **9** | **DL Gen** | **5** | **250x50** | **5/2/2/No/No\| OR** | **0.8514** | **0.7949** | **0.8592** | **0.8270** | **0.8164** | **244** | **40** | **8** | **31** | **48** |
| 9 No DA | DL Gen | 5 | 250x50 | No/No/No/No/No\| - | 0.6378 | 0.7949 | 0.6162 | 0.7055 | 0.7768 | 175 | 109 | 8 | 31 | 117 |

Abbreviations: %, percentage variation; NPP, new positive patients created; NNP, new negative patients created; F, vertical flip applied; R, random rotation applied; Range, variation range used in data augmentation (DA); OR, original range; LR, limited range; B, binary variables; Vars, number of variables.

**Figure S5**: Calibration analysis of DL Gen 8 and baseline models on the test set. (A) Reliability diagram showing the observed frequency of GDM versus the mean predicted probability within each of 10 equal-frequency bins for DL Gen 8, DL 8, and Trad 8 (SVM). The dashed line indicates perfect calibration. Summary metrics, including the Brier score, calibration slope, calibration intercept, and Expected Calibration Error (ECE), are shown in the inset. (B) Distribution of predicted probabilities in the test set for each model, showing that most predictions are concentrated in the 0.00–0.20 probability range.

**Figure S6**: DCA of DL Gen 8 and baseline models on the test set. Net benefit is shown across the evaluated range of threshold probabilities for DL Gen 8, DL 8, and Trad 8 (SVM), together with the Treat All and Treat None strategies. Higher net benefit indicates greater potential clinical utility at a given threshold probability.

**Figure S7**: Mapping between genotype positions and clinical variables in the input image of the DL Gen model (Model 7). The genetic algorithm assigns each of the 50 columns of the 250 × 50 image to one of the five continuous variables, allowing multiple positions per variable. Color encoding indicates the variable assigned to each column. This mapping facilitates interpretation of the spatial attention patterns generated by Grad-CAM (Figure 9) in relation to the clinical variables.

**Table S8**: Comparison of AUCROC and Recall Macro (RM) performance in validation set and test set of models DL Gen 1 to 9.

| **Model** | **Val AUC** | **Test AUC** | **Δ AUC** | **Val RM** | **Test RM** | **Δ RM** |
| --- | --- | --- | --- | --- | --- | --- |
| DL Gen 1 | 0.8272 | 0.8414 | -0.0142 | 0.8245 | 0.7747 | +0.0498 |
| DL Gen 2 | 0.8136 | 0.8329 | -0.0193 | 0.8209 | 0.7794 | +0.0414 |
| DL Gen 3 | 0.8149 | 0.8318 | -0.0169 | 0.8064 | 0.7913 | +0.0151 |
| DL Gen 4 | 0.8048 | 0.8519 | -0.0471 | 0.8028 | 0.8454 | -0.0426 |
| DL Gen 5 | 0.7931 | 0.8422 | -0.0491 | 0.7956 | 0.8360 | -0.0404 |
| DL Gen 6 | 0.7912 | 0.8470 | -0.0558 | 0.8064 | 0.8338 | -0.0274 |
| DL Gen 7 | 0.7991 | 0.8425 | -0.0434 | 0.8028 | 0.8333 | -0.0305 |
| DL Gen 8 | 0.7292 | 0.8331 | -0.1039 | 0.7883 | 0.8434 | -0.0551 |
| DL Gen 9 | 0.7314 | 0.8164 | -0.0850 | 0.7811 | 0.8271 | -0.0460 |

**Table S9:** Bootstrap 95% confidence intervals (1000 iterations) for test-set metrics.

| Model | Sensitivity | Specificity | PPV | AUC-ROC |
| --- | --- | --- | --- | --- |
| DL Gen 1 | 1.000 [1.000, 1.000] | 0.549 [0.493, 0.606] | 0.234 [0.169, 0.300] | 0.841 [0.791, 0.888] |
| DL 1 | 1.000 [1.000, 1.000] | 0.331 [0.273, 0.382] | 0.170 [0.119, 0.219] | 0.788 [0.719, 0.855] |
| Trad 1 | 1.000 [1.000, 1.000] | 0.317 [0.264, 0.371] | 0.167 [0.123, 0.213] | 0.819 [0.757, 0.885] |
| DL Gen 2 | 0.974 [0.906, 1.000] | 0.585 [0.529, 0.640] | 0.244 [0.181, 0.317] | 0.833 [0.777, 0.883] |
| DL 2 | 0.974 [0.912, 1.000] | 0.570 [0.512, 0.628] | 0.237 [0.175, 0.302] | 0.785 [0.716, 0.844] |
| Trad 2 | 0.974 [0.909, 1.000] | 0.493 [0.435, 0.552] | 0.209 [0.153, 0.270] | 0.800 [0.721, 0.867] |
| DL Gen 3 | 0.949 [0.875, 1.000] | 0.634 [0.580, 0.688] | 0.262 [0.191, 0.338] | 0.832 [0.774, 0.884] |
| DL 3 | 0.949 [0.875, 1.000] | 0.588 [0.523, 0.649] | 0.240 [0.176, 0.307] | 0.810 [0.735, 0.875] |
| Trad 3 | 0.949 [0.871, 1.000] | 0.560 [0.500, 0.621] | 0.228 [0.170, 0.294] | 0.823 [0.753, 0.882] |
| DL Gen 4 | 0.923 [0.829, 1.000] | 0.768 [0.714, 0.816] | 0.353 [0.263, 0.443] | 0.852 [0.792, 0.907] |
| DL 4 | 0.923 [0.838, 1.000] | 0.609 [0.552, 0.669] | 0.245 [0.181, 0.320] | 0.785 [0.720, 0.845] |
| Trad 4 | 0.923 [0.838, 1.000] | 0.570 [0.514, 0.628] | 0.228 [0.163, 0.291] | 0.823 [0.748, 0.889] |
| DL Gen 5 | 0.897 [0.793, 0.978] | 0.775 [0.727, 0.823] | 0.354 [0.256, 0.448] | 0.842 [0.773, 0.900] |
| DL 5 | 0.897 [0.788, 0.976] | 0.658 [0.604, 0.714] | 0.265 [0.188, 0.338] | 0.811 [0.732, 0.878] |
| Trad 5 | 0.897 [0.792, 0.977] | 0.627 [0.567, 0.682] | 0.248 [0.181, 0.326] | 0.820 [0.746, 0.883] |
| DL Gen 6 | 0.872 [0.763, 0.970] | 0.796 [0.744, 0.841] | 0.370 [0.269, 0.468] | 0.847 [0.779, 0.902] |
| DL 6 | 0.872 [0.763, 0.973] | 0.697 [0.644, 0.753] | 0.283 [0.207, 0.362] | 0.809 [0.727, 0.883] |
| Trad 6 | 0.872 [0.773, 0.972] | 0.662 [0.608, 0.716] | 0.262 [0.188, 0.338] | 0.816 [0.746, 0.879] |
| DL Gen 7 | 0.846 [0.711, 0.944] | 0.820 [0.775, 0.865] | 0.393 [0.283, 0.500] | 0.842 [0.773, 0.898] |
| DL 7 | 0.846 [0.727, 0.955] | 0.761 [0.714, 0.811] | 0.327 [0.236, 0.424] | 0.803 [0.717, 0.877] |
| Trad 7 | 0.846 [0.737, 0.950] | 0.690 [0.630, 0.742] | 0.273 [0.197, 0.357] | 0.814 [0.742, 0.882] |
| DL Gen 8 | 0.821 [0.692, 0.929] | 0.866 [0.825, 0.905] | 0.457 [0.339, 0.580] | 0.833 [0.760, 0.899] |
| DL 8 | 0.821 [0.690, 0.931] | 0.771 [0.721, 0.817] | 0.330 [0.239, 0.423] | 0.818 [0.735, 0.884] |
| Trad 8 | 0.821 [0.687, 0.939] | 0.746 [0.695, 0.794] | 0.308 [0.219, 0.402] | 0.813 [0.738, 0.876] |
| DL Gen 9 | 0.795 [0.649, 0.912] | 0.859 [0.818, 0.898] | 0.437 [0.319, 0.548] | 0.816 [0.733, 0.885] |
| DL 9 | 0.795 [0.657, 0.915] | 0.799 [0.754, 0.843] | 0.352 [0.258, 0.446] | 0.823 [0.743, 0.895] |
| Trad 9 | 0.795 [0.656, 0.915] | 0.732 [0.683, 0.783] | 0.290 [0.204, 0.382] | 0.814 [0.736, 0.879] |

**Table S10**: Paired bootstrap tests (1000 iterations) for the difference in specificity and PPV between DL Gen and each baseline (DL without GA and traditional ML) at matched sensitivity levels on the test set (n=323, 39 GDM cases).

| **Comparison** | **Sens** | **Spec A** | **Spec B** | **Δ Spec [95% CI]** | **p (Spec)** | **PPV A** | **PPV B** | **Δ PPV [95% CI]** | **p (PPV)** |
| --- | --- | --- | --- | --- | --- | --- | --- | --- | --- |
| DL Gen 1 vs DL 1 | 1.000 | 0.549 | 0.331 | +0.218 [+0.154, +0.286] | <0.001 | 0.234 | 0.170 | +0.063 [+0.040, +0.092] | <0.001 |
| DL Gen 2 vs DL 2 | 0.974 | 0.585 | 0.570 | +0.014 [-0.050, +0.078] | 0.658 | 0.244 | 0.237 | +0.006 [-0.027, +0.038] | 0.734 |
| DL Gen 3 vs DL 3 | 0.949 | 0.634 | 0.588 | +0.046 [-0.011, +0.101] | 0.116 | 0.262 | 0.240 | +0.022 [-0.010, +0.056] | 0.216 |
| DL Gen 4 vs DL 4 | 0.923 | 0.768 | 0.609 | +0.158 [+0.107, +0.212] | <0.001 | 0.353 | 0.245 | +0.108 [+0.059, +0.157] | <0.001 |
| DL Gen 5 vs DL 5 | 0.897 | 0.775 | 0.658 | +0.116 [+0.059, +0.180] | <0.001 | 0.354 | 0.265 | +0.088 [+0.033, +0.154] | 0.002 |
| DL Gen 6 vs DL 6 | 0.872 | 0.796 | 0.697 | +0.099 [+0.041, +0.154] | 0.004 | 0.370 | 0.283 | +0.086 [+0.017, +0.151] | 0.012 |
| DL Gen 7 vs DL 7 | 0.846 | 0.820 | 0.761 | +0.060 [+0.007, +0.110] | 0.020 | 0.393 | 0.327 | +0.066 [-0.002, +0.134] | 0.056 |
| **DL Gen 8 vs DL 8** | **0.821** | **0.866** | **0.771** | **+0.095 [+0.052, +0.142]** | **<0.001** | **0.457** | **0.330** | **+0.127 [+0.054, +0.206]** | **<0.001** |
| DL Gen 9 vs DL 9 | 0.795 | 0.859 | 0.799 | +0.060 [+0.018, +0.103] | 0.002 | 0.437 | 0.352 | +0.084 [+0.014, +0.163] | 0.014 |
| DL Gen 1 vs Trad 1 | 1.000 | 0.549 | 0.317 | +0.232 [+0.164, +0.300] | <0.001 | 0.234 | 0.167 | +0.066 [+0.041, +0.097] | <0.001 |
| DL Gen 2 vs Trad 2 | 0.974 | 0.585 | 0.493 | +0.092 [+0.029, +0.156] | 0.004 | 0.244 | 0.209 | +0.035 [+0.009, +0.065] | 0.010 |
| DL Gen 3 vs Trad 3 | 0.949 | 0.634 | 0.560 | +0.074 [+0.018, +0.131] | 0.002 | 0.262 | 0.228 | +0.034 [+0.005, +0.069] | 0.024 |
| DL Gen 4 vs Trad 4 | 0.923 | 0.768 | 0.570 | +0.197 [+0.142, +0.257] | <0.001 | 0.353 | 0.228 | +0.125 [+0.081, +0.181] | <0.001 |
| DL Gen 5 vs Trad 5 | 0.897 | 0.775 | 0.627 | +0.148 [+0.102, +0.197] | <0.001 | 0.354 | 0.248 | +0.105 [+0.064, +0.157] | <0.001 |
| DL Gen 6 vs Trad 6 | 0.872 | 0.796 | 0.662 | +0.134 [+0.082, +0.184] | <0.001 | 0.370 | 0.262 | +0.108 [+0.061, +0.162] | <0.001 |
| DL Gen 7 vs Trad 7 | 0.846 | 0.820 | 0.690 | +0.130 [+0.085, +0.176] | <0.001 | 0.393 | 0.273 | +0.120 [+0.066, +0.179] | <0.001 |
| **DL Gen 8 vs Trad 8** | **0.821** | **0.866** | **0.746** | **+0.120 [+0.072, +0.171]** | **<0.001** | **0.457** | **0.308** | **+0.149 [+0.077, +0.229]** | **<0.001** |
| DL Gen 9 vs Trad 9 | 0.795 | 0.859 | 0.732 | +0.127 [+0.079, +0.178] | <0.001 | 0.437 | 0.290 | +0.147 [+0.074, +0.236] | <0.001 |

For each pair (DL Gen model M vs baseline model M), both models are evaluated at their respective operating thresholds selected to achieve the same sensitivity level (values in the "Sens" column). Δ Spec = Spec (DL Gen) − Spec (baseline); Δ PPV = PPV (DL Gen) − PPV (baseline). Positive values indicate advantage of DL Gen. CIs and p-values were computed via paired bootstrap resampling with 1000 iterations. Two-sided p-values were computed as 2 × min(proportion of bootstrap iterations with negative difference, proportion with positive difference). Rows corresponding to the recommended Model 8 are shown in bold.

**Table S11:** Distribution of participants with early-pregnancy 1TFG ≥ 92 mg/dL across data splits.

| **Split** | **Total (n)** | **1TFG ≥ 92 (n, %)** | **GDM among 1TFG ≥ 92 (n, %)** |
| --- | --- | --- | --- |
| Training | 1,127 | 99 (8.8%) | 49 (49.5%) |
| Validation | 161 | 13 (8.1%) | 6 (46.2%) |
| Test | 323 | 28 (8.7%) | 14 (50.0%) |
| **Total** | **1,611** | **140 (8.7%)** | **69 (49.3%)** |

**Table S12:** Sensitivity analysis. Test-set performance of DL Gen models 4, 6, and 8 on the original data (full test set, n=323) and on the filtered subset (n=295, patients with 1TFG ≥ 92 mg/dL excluded from all splits).

| **Model** | **Configuration** | **Sens** | **Spec** | **AUC-ROC** | **TN** | **FP** | **FN** | **TP** | **FP+FN** |
| --- | --- | --- | --- | --- | --- | --- | --- | --- | --- |
| **Model 4** | Original (full test set) | 0.923 | 0.768 | 0.852 | 218 | 66 | 3 | 36 | 69 |
|  | Retrained, fixed hyperparameters | 0.920 | 0.541 | 0.773 | 146 | 124 | 2 | 23 | 126 |
|  | Retrained, re-selected hyperparameters | 0.920 | 0.633 | 0.755 | 171 | 99 | 3 | 22 | 102 |
| **Model 6** | Original (full test set) | 0.872 | 0.796 | 0.847 | 226 | 58 | 5 | 34 | 63 |
|  | Retrained, fixed hyperparameters | 0.880 | 0.641 | 0.764 | 173 | 96 | 3 | 22 | 99 |
|  | Retrained, re-selected hyperparameters | 0.880 | 0.741 | 0.790 | 200 | 70 | 3 | 22 | 73 |
| **Model 8** | Original (full test set) | 0.821 | 0.866 | 0.833 | 246 | 38 | 7 | 32 | 45 |
|  | Retrained, fixed hyperparameters | 0.800 | 0.426 | 0.720 | 115 | 155 | 5 | 20 | 160 |
|  | Retrained, re-selected hyperparameters | 0.800 | 0.796 | 0.810 | 215 | 55 | 5 | 20 | 60 |

"Original" refers to the model trained on the full training set (1,127 patients) and evaluated on the full test set (n=323, 39 GDM cases). "Retrained" refers to the model trained on the filtered training set (1,028 patients, patients with 1TFG ≥ 92 excluded), using the same GA-optimized genotype identified on the original data. "Fixed hyperparameters" retains the CNN hyperparameters selected for the original data. "Re-selected hyperparameters" reselects the CNN hyperparameters based on the filtered validation set (n=148, 17 GDM cases). All retrained models are evaluated on the filtered test set (n=295, 25 GDM cases). Reported sensitivity is the closest available operating point to the target sensitivity of the corresponding original model.

**Table S13**: Comparison of test AUC-ROC between GA-optimized Model 8 and 50 independently sampled random-genotype configurations. Summary statistics.

| **Configuration** | **Test AUC-ROC** | **N models** |
| --- | --- | --- |
| **GA-optimized (Model 8)** | **0.8330** | **1** |
| Random genotypes — median | 0.7230 | 50 |
| Random genotypes — mean (SD) | 0.7251 (0.0259) | 50 |
| Random genotypes — IQR | [0.7115, 0.7435] | 50 |
| Random genotypes — range | [0.6672, 0.7774] | 50 |
| Random genotypes — 95% percentile range | [0.6740, 0.7734] | 50 |
| GA gap over best random | +0.056 (+5.6 pp) | — |
| GA gap over median random | +0.110 (+11.0 pp) | — |
| Percentile of GA in random dist. | 100% (0/50 reached GA) | — |

**Table S14:** Individual test AUC-ROC values for the 50 random-genotype configurations.

| **#** | **AUC** | **#** | **AUC** | **#** | **AUC** | **#** | **AUC** |
| --- | --- | --- | --- | --- | --- | --- | --- |
| 1 | 0.7774 | 14 | 0.7434 | 27 | 0.7199 | 40 | 0.7057 |
| 2 | 0.7748 | 15 | 0.7429 | 28 | 0.7189 | 41 | 0.7030 |
| 3 | 0.7686 | 16 | 0.7427 | 29 | 0.7188 | 42 | 0.7017 |
| 4 | 0.7667 | 17 | 0.7402 | 30 | 0.7185 | 43 | 0.6941 |
| 5 | 0.7633 | 18 | 0.7385 | 31 | 0.7183 | 44 | 0.6928 |
| 6 | 0.7565 | 19 | 0.7368 | 32 | 0.7170 | 45 | 0.6898 |
| 7 | 0.7496 | 20 | 0.7356 | 33 | 0.7152 | 46 | 0.6896 |
| 8 | 0.7489 | 21 | 0.7324 | 34 | 0.7148 | 47 | 0.6831 |
| 9 | 0.7484 | 22 | 0.7278 | 35 | 0.7142 | 48 | 0.6793 |
| 10 | 0.7481 | 23 | 0.7272 | 36 | 0.7137 | 49 | 0.6724 |
| 11 | 0.7449 | 24 | 0.7255 | 37 | 0.7135 | 50 | 0.6672 |
| 12 | 0.7447 | 25 | 0.7235 | 38 | 0.7108 |  |  |
| 13 | 0.7435 | 26 | 0.7224 | 39 | 0.7082 |  |  |

Random genotypes were independently sampled as length-50 vectors of variable identifiers drawn uniformly from {0, 1, 2, 3, 4}. Each model was trained using the same hyperparameters, data augmentation, and training procedure as Model 8. AUC-ROC is computed on the same test set (n=323; 39 GDM cases). Individual values are sorted in descending order.

**Table S15:** Performance of univariate baseline using 1TFG alone compared with Model 8 (multivariable DL Gen with 5 continuous variables). Comparison at operating sensitivity 0.82

| **Metric** | **Univariate 1TFG (LR)** | **DL Gen 8 (5 variables)** | **Δ** |
| --- | --- | --- | --- |
| AUC-ROC [95% CI] | 0.785 [0.705, 0.862] | 0.833 [0.760, 0.899] | +0.048 |
| Sensitivity | 0.821 | 0.821 | 0 |
| Specificity [95% CI] | 0.539 [0.478, 0.593] | 0.866 [0.825, 0.905] | +32.7 pp |
| PPV [95% CI] | 0.196 [0.136, 0.261] | 0.457 [0.339, 0.580] | +26.1 pp |
| TP / FN | 32 / 7 | 32 / 7 | 0 |
| FP / TN | 131 / 153 | 38 / 246 | -93 FP |

**Table S16:** Performance of the trivial threshold rule '1TFG ≥ x mg/dL' at various thresholds

| **Threshold (mg/dL)** | **Sensitivity** | **Specificity** | **PPV** | **TN** | **FP** | **FN** | **TP** | **FP+FN** |
| --- | --- | --- | --- | --- | --- | --- | --- | --- |
| 80 | 0.744 | 0.648 | 0.225 | 184 | 100 | 10 | 29 | 110 |
| 85 | 0.590 | 0.824 | 0.315 | 234 | 50 | 16 | 23 | 66 |
| 88 | 0.462 | 0.912 | 0.419 | 259 | 25 | 21 | 18 | 46 |
| 90 | 0.385 | 0.930 | 0.429 | 264 | 20 | 24 | 15 | 44 |
| **92 (IADPSG)** | **0.359** | **0.951** | **0.500** | **270** | **14** | **25** | **14** | **39** |
| 95 | 0.231 | 0.989 | 0.750 | 281 | 3 | 30 | 9 | 33 |

The univariate LR baseline uses Logistic Regression with balanced class weights on standardized 1TFG values. The threshold-based baseline predicts positive if 1TFG ≥ x mg/dL. Both univariate methods achieve the same AUC-ROC (0.785) as they are monotonic transformations of the same variable. The IADPSG threshold (92 mg/dL) corresponds to the fasting glucose cutoff used in the OGTT-based GDM diagnosis. Model 8 metrics are as reported in Table 3. 95% confidence intervals were computed via bootstrap resampling (1000 iterations). Test set n=323 with 39 GDM cases.

**Table S17:** Comparison of Model 8 using two different backbone initialization strategies: ImageNet-pretrained weights (original) and randomly initialized weights, with the initialization variance scaled according to the number of input and output units in each layer.

| **Metrics** | **Model 8 ImageNet Weight (original)** | **Model 8 Random Weights** | **Δ** |
| --- | --- | --- | --- |
| Accuracy | 0.860 | 0.839 | -0.021 |
| Sensitivity | 0.821 | 0.821 | 0.000 |
| Specificity | 0.866 | 0.842 | -0.024 |
| Recall Macro | 0.844 | 0.831 | -0.013 |
| AUC-ROC | 0.833 | 0.830 | -0.003 |
| PPV | 0.457 | 0.416 | -0.041 |
| TN | 246 | 239 | -7 |
| FP | 38 | 45 | +7 |
| FN | 7 | 7 | 0 |
| TP | 32 | 32 | 0 |

**Table S18:** Comparison of variable importance rankings between Grad-CAM (DL Gen 8), Logistic Regression, and XGBoost proxy models.

| **Variable** | **Grad-CAM (TP-TN contrast)** | **LR permutation Δ AUC [95% CI]** | **XGB permutation Δ AUC [95% CI]** | **Column position (mean)** |
| --- | --- | --- | --- | --- |
| 1t_fg | +0.080 | +0.186 [+0.124, +0.258] | +0.167 [+0.094, +0.225] | 22.6 |
| Mat_wei1 | -0.090 | +0.004 [-0.005, +0.014] | +0.013 [-0.026, +0.053] | 28.0 |
| BMI_1 | -0.006 | +0.022 [+0.002, +0.043] | +0.041 [-0.006, +0.082] | 23.4 |
| Age | -0.006 | +0.044 [+0.005, +0.086] | +0.049 [-0.007, +0.110] | 26.3 |
| Parity | +0.022 | +0.006 [-0.002, +0.015] | +0.007 [-0.006, +0.025] | 23.8 |

*Grad-CAM values are the mean TP-TN contrast; positive values indicate attribution toward the positive class (GDM), negative values toward the negative class (non-GDM). LR and XGB permutation importance values are the mean drop in AUC-ROC (over 100 permutations) with 95% confidence intervals. Column position is the average position (0-49) of the columns to which the genetic algorithm assigned each variable in the image representation.*

**Table S19:** Spearman correlations between variable importance rankings and correlations between importance and column position.

| **Comparison** | **Spearman ρ** | **p-value** |
| --- | --- | --- |
| LR vs XGB (ranking agreement) | +0.900 | 0.037 |
| LR vs Grad-CAM (ranking agreement) | -0.300 | 0.624 |
| XGB vs Grad-CAM (ranking agreement) | -0.100 | 0.873 |
|  |  |  |
| LR importance vs column position | -0.700 | 0.188 |
| XGB importance vs column position | -0.500 | 0.391 |
| Grad-CAM importance vs column position | +0.205 | 0.741 |

*Ranking agreement is computed using Spearman correlation between variable ranks. Importance vs column position is computed using Spearman correlation between the importance values and the average column position assigned by the genetic algorithm.*
